# Tumor suppressor SLC9A2 inhibits colorectal cancer metastasis and reverses immunotherapy resistance by suppressing angiogenesis

**DOI:** 10.1186/s13046-025-03422-7

**Published:** 2025-06-05

**Authors:** Zizhen Zhang, Shengde Liu, Ting Xu, Nan Chen, Cheng Liu, Hong Yang, Yuanmeng Shi, Zhiwei Li, Xujiao Feng, Yanhong Yao, Xiaorui Duan, Gehan Xu, Cheng Zhang, Zhenghang Wang, Jian Li, Lin Shen

**Affiliations:** 1 Key laboratory of Carcinogenesis and Translational Research (Ministry of Education/Beijing), Department of Gastrointestinal Oncology, Peking University Cancer Hospital & Institute, Beijing, 100142 China; 2 Key Laboratory of Carcinogenesis and Translational Research (Ministry of Education/Beijing), Gastrointestinal Cancer Center, Unit III, Peking University Cancer Hospital & Institute, Beijing, 100142 China; 3 Key Laboratory of Carcinogenesis and Translational Research (Ministry of Education/Beijing), Gastrointestinal Cancer Center, Unit IV, Peking University Cancer Hospital & Institute, Beijing, 100142 China; 4 Department of Gastrointestinal Oncology, Inner Mongolia Cancer Center, Peking University Cancer Hospital (Lnner Mongolia Campus)/Affiliated Cancer Hospital of Inner Mongolia Medical University, Hohhot, 010020 China; 5 Department of Gastrointestinal Surgery, Inner Mongolia Cancer Center, Peking University Cancer Hospital (Lnner Mongolia Campus)/Affiliated Cancer Hospital of Inner Mongolia Medical University, Hohhot, 010020 China

**Keywords:** Colorectal Cancer, SLC9A2, Angiogenesis, Liver metastasis, immunotherapy

## Abstract

**Background:**

Colorectal cancer (CRC) is a common and aggressive malignancy that frequently metastasizes to the liver, presenting significant therapeutic challenges. Despite its clinical importance, the mechanisms underlying CRC liver metastasis and resistance to immune therapy remain poorly understood. In this study, we aimed to investigate the molecular mechanisms driving CRC metastasis using a novel approach, which includes the establishment of highly metastatic CRC cell lines.

**Methods:**

To explore the role of specific genes in CRC liver metastasis, we developed two highly metastatic CRC cell lines (LoVo-Hm and HCT116-Hm) by applying sustained selective pressure to primary CRC cells. RNA sequencing was performed to identify differentially expressed genes in these metastatic cells. Additionally, we conducted assays for cell migration, invasion, angiogenesis, and ELISA to evaluate VEGFA production, all to confirm the functional role of SLC9A2. Our findings were further validated in human CRC tissue samples and publicly available datasets to assess the clinical relevance of the identified targets.

**Results:**

Our analysis revealed a significant downregulation of SLC9A2 in the highly metastatic CRC cell lines. Mechanistically, we found that SLC9A2 inhibits epithelial-mesenchymal transition (EMT) and metastasis by suppressing the STAT3 signaling pathway. Moreover, SLC9A2 reduces VEGFA secretion, normalizing tumor vasculature and reshaping the tumor microenvironment (TME), which ultimately enhances anti-tumor immunity. Comparative analysis of CRC tissue samples showed reduced SLC9A2 expression in tumor tissues compared to adjacent normal tissues, with a negative correlation to TNM staging. Importantly, higher SLC9A2 expression was associated with better treatment responses in immunotherapy cohorts.

**Conclusion:**

These findings highlight the critical role of SLC9A2 in regulating metastasis, angiogenesis, and TME remodeling in CRC. By modulating the STAT3 pathway and tumor vasculature, SLC9A2 emerges as a potential prognostic biomarker and therapeutic target. Targeting SLC9A2 may enhance immune responses and improve treatment outcomes in CRC, offering a promising avenue for future therapeutic strategies.

**Supplementary Information:**

The online version contains supplementary material available at 10.1186/s13046-025-03422-7.

## Significance

SLC9A2 loss enhances colorectal cancer metastasis and immunotherapy resistance by inhibiting STAT3 signaling and angiogenesis, establishing it as a key tumor suppressor and biomarker for colorectal cancer.

**Supplementary Information:**

The online version contains supplementary material available at 10.1186/s13046-025-03422-7.

## Introduction

Colorectal cancer (CRC) ranks as the third most prevalent malignancy worldwide, with its incidence and mortality rates placing it third and second, respectively, among all cancers [1, 2]. Tumor metastasis remains the leading cause of death among CRC patients. Clinically, more than 60% of patients with CRC present with liver metastases, of which more than 80-90% are not initially resectable [3, 4]. The median survival for without surgical treatment CRC patients with liver metastases is a mere 6.9 months, with a five-year survival rate of less than 5% [5]. Despite the promising results of immunotherapy in CRC, its efficacy in cases of liver metastasis remains minimal [6]. A comprehensive understanding of the molecular mechanisms underlying CRC liver metastasis is essential for advancing potential therapeutic interventions.

Tumors have been considered the result of neo-Darwinian evolution processes within tissues [7, 8]. In terms of evolutionary biology, the transformation of normal cells into tumors necessitates a series of genetic and epigenetic alterations [9, 10]. Within tumors, cancer cells constitute a heterogeneous population, comprising both tumor-initiating cells and differentiated cells that exhibit distinct morphological and functional characteristics [11]. Evidence suggests that selective pressures within the tumor microenvironment could drive intratumoral heterogeneity [12, 13]. Likewise, during the metastatic progression of primary colorectal cancer to the liver, these selective pressures contribute to significant heterogeneity between primary and metastatic lesions [14, 15]. Therefore, elucidating the mechanisms that underpin colorectal cancer metastasis and identifying therapeutic targets for liver metastases are critical for the effective management of this disease.

SLC9A2, also known as NHE2, encodes a member of the Na^+^/H^+^ exchanger (NHE) protein family [16]. The protein is localized to the apical membrane and is prone to lysosomal degradation, characterizing it as a protein with a short half-life [17]. SLC9A2 participates in sodium ion transport by exchanging intracellular protons for extracellular sodium ions, thereby helping to regulate cellular pH and neutralize metabolic acids while counteracting adverse environmental conditions [18]. Studies have shown that SLC9A2 is expressed not only in gastrointestinal epithelial cells but also in the kidneys and endometrium [17]. However, the expression changes of SLC9A2 during the metastatic progression of tumors, as well as its influence on the metastatic potential of CRC and the effects of CRC liver metastases on immunotherapy, remain unexplored. Further research is needed to clarify the precise role and underlying mechanisms of SLC9A2 in the malignant progression of CRC.

Here, we employed in vitro and in vivo pressure screening to identify subclones of tumor cells with high metastatic and invasive capacities. Our findings reveal a loss of SLC9A2 protein expression during the metastatic progression of CRC. The absence of SLC9A2 enhances the migration and invasion abilities of tumor cells, and its loss in CRC liver metastases contributes to the development of resistance to immunotherapy. Mechanistically, downregulation of SLC9A2 activates the STAT3 pathway in tumor cells, promoting their migration and invasion. Furthermore, activation of the STAT3 pathway leads to increased secretion of vascular endothelial growth factor A (VEGFA), enhancing angiogenesis and further facilitating tumor metastasis. Collectively, our study elucidates for the first time the intracellular mechanisms of the SLC9A2/STAT3/VEGFA axis in CRC and highlights its significant therapeutic potential in translational medicine.

## Materials and methods

### Clinical specimens

All clinical specimens from CRC patients, including paraffin-embedded sections, fresh-frozen tissues, blood samples, and prognostic data, were collected at Beijing Cancer Hospital. Specimens were obtained with informed consent from patients, and the study protocol was approved by the ethics committee of Peking University Cancer Hospital (Ethics Approval Number: 2023YJZ16).

### Cell lines

The LoVo, HCT116, Human Umbilical Vein Endothelial Cells (HUVECs) and MC38 were obtained from the National Infrastructure of Cell Line Resource (Beijing, China). LoVo, HCT116 cells were cultured in DMEM medium (Gibco) supplemented with 10% fetal bovine serum (FBS) (Gibco) and 100 U/ml penicillin-streptomycin at 37 °C with 5% CO_2_. HUVECs and MC38 cells were maintained in 1640 medium supplemented with 10% FBS and 100 U/ml penicillin/streptomycin. The cells were cultured to approximately 90% confluence, harvested by digestion, resuspended in CELLSAVING^TM^ (C40100, New Cell & Molecular Biotech), aliquoted into cryovials (NEST Biotechnology Co., Ltd.) and stored at -80°C.

### Animal use and care

All animal experiments were conducted in accordance with the guidelines set forth by the Institutional Animal Care and Use Committee of Beijing Cancer Hospital (Ethics Approval Number: EAEC-2023-13). BALB/c nude, C57BL/6J, and NOD.CB17-Prkdc scid /NcrCrl (NOD-SCID) mice were procured from Beijing Vital River Laboratory Animal Technology, China. The mice were housed under specific pathogen-free (SPF) conditions in ventilated cages with controlled environmental parameters, including a 12-hour light/dark cycle, constant temperature, and humidity. They had ad libitum access to enriched water and food.

### Antibodies and reagents

Antibodies against β-actin (HA722023), GAPDH (ET1601-4), Lamin B1 (ET1606-27), CDX2 (ET1605-4), CK7 (ET1609-62), CK20 (ET7110-54), ki67 (HA721115), β-catenin (ET1601-5), E-Cadherin (ET1607-75), N-Cadherin (ET1607-37), Claudin-1 (ER1906-37), Snail (ER1706-22), Vimentin (ET1610-39), and CD31 (M1511-8) were obtained from Hangzhou HuaAn Biotechnology. Antibodies against STAT3 (#9139) and p-STAT3^Y705^ (#9131) were provided by Cell Signaling Technology. The SLC9A2 antibody was purchased from Thermo Fisher (PA5-80034). In all experiments, plasmids were constructed using Gibson assembly cloning techniques as described previously [19]. Full-length human SLC9A2 was amplified from LoVo cell cDNA with the forward primer 5′- ATGGAACCACTGGGCAACTGG − 3′ and reverse primer 5′- AGGCTTCTCACTCCCAAATCGG − 3′. Full-length mouse Slc9a2 was amplified from MC38 cell cDNA using the forward primer 5′- ATGGGCCCCCGGGGCACCGCGCA − 3′ and reverse primer 5′- TGGCTTTTCATTGCCAAGGCGGCCT − 3′. The p-STAT3^Y705^ inhibitor Stattic (MCE, HY-13818) and the JAK1/2 inhibitor Ruxolitinib (MCE, HY-50856) were utilized in this study.

### Western blot

Proteins of Cells or PDOs were lysed and harvested then Western blot analysis was performed according to the standard protocol. In brief, proteins were lysed in RIPA lysis buffer on ice for 30 min. Following separation by SDS/PAGE, proteins were transferred onto 0.45 μm polyvinylidene difluoride (PVDF) membranes and incubated overnight with primary antibodies. Subsequently, membranes were probed with HRP-conjugated secondary antibodies and visualized using enhanced chemiluminescence.

### Cell migration and invasion assay

For the migration assay, CRC cells (ranging from 1 × 10^5^ to 2 × 10^5^) were placed in the upper chamber of an 8 μm Transwell insert (Corning Costar). Prior to cell seeding, 50 µL of diluted Matrigel solution (CAT#356234; Corning) was added to the upper chamber for invasion experiments. Next, 700 µL of culture medium containing 15% FBS was added to the lower chamber. After incubation for 24–48 h, cells were fixed with 4% paraformaldehyde and stained with crystal violet. the cells that migrated to the lower side of the chambers were photographed using a Leica microscope.

### Wound healing assay

The CRC cells were seeded into a 6-well plate to achieve an approximate confluence of 80%. Upon reaching about 95% confluence, three vertical scratches were created in each well using a sterile blue pipette tip. The plate was subsequently washed once with PBS and replenished with 2% FBS-supplemented DMEM or conditioned medium. The scratches were located under a microscope, and images were captured at various time points. Finally, ImageJ software was utilized to perform quantitative image analysis of wound healing ratio at specified locations across different time intervals.

### 5-Ethynyl-2′-Deoxyuridine (EdU) assay

The EdU-594 Cell Proliferation Assay Kit (Beyotime, C0078S) was employed to assess cell proliferation according to the standard protocol. HUVEC cells were cultured overnight in a 6-well plate. The next day, 2× EdU working solution was added in equal volume to achieve a final concentration of 10 µM, and cells were cultured for an additional 2 h at 37 °C. After EdU labeling, cells were fixed with 1 ml of 4% paraformaldehyde at room temperature (RT) for 15 min. Subsequently, cells were washed three times with 1 ml of washing buffer and then incubated with 1 ml of permeabilization solution at RT for 15 min. After additional washing, 0.5 ml of Click reaction solution was added to each well, and the plate was gently shaken for uniform distribution. Cells were then incubated in the dark at room temperature for 30 min. Following removal of the Click reaction solution, cells were washed three times with washing buffer. Finally, cells were examined and imaged using a fluorescence microscope with an excitation wavelength of 594 nm.

### ELISA assay

Approximately 100 mg of subcutaneous tumor tissue from mice underwent five freeze-thaw cycles in liquid nitrogen. Then, 1 ml of PBS was added per 100 mg of tissue, and samples were homogenized. The homogenate was centrifuged at 8,000 rpm for 10 min, and the supernatant was collected for ELISA analysis. Mouse granzyme B (SEKM-0088), IFN-γ (SEKM-0031) and VEGFA (SEKM-0039) ELISA kits were obtained from Beijing Solarbio Science & Technology Co., Ltd.

### Conditional medium (CM) preparation

CRC cells were seeded in 6-well plates to achieve approximately 70% confluency within 24 h. Subsequently, cells were transfected with either siRNA or plasmids to induce knockdown or overexpression of SLC9A2. Following transfection, cells were washed three times with PBS and maintained in serum-free medium for an additional 24 h. Cell culture supernatants were collected, centrifuged at 2000 g at 4 °C for 10 min, and either used immediately or stored at -80 °C for subsequent analysis.

### HUVEC cells tubule formation assay

HUVEC cells were seeded in 12-well plates and allowed to reach approximately 70% confluence within 24 h. Subsequently, cells were stimulated with specific conditioned medium for 24 h. Following stimulation, a mixture of 35 µL of Matrigel (BD Matrigel Matrix, Cat. 356234) and 15 µL of serum-free DMEM was coated on the bottom of a 96-well plate and polymerized at 37 °C for 30 min. Then, 200 µL of CM containing 4 × 10^4^ HUVEC cells was added to the polymerized Matrigel and incubated for 6 h at 37 °C. Finally, the capillary tubes were observed and captured using an optical microscope.

### Generation of patient derived organoids (PDOs)

Fresh CRC surgical specimens were placed in collection tubes containing tissue preservation solution (MB-0818L04L) and transported to the laboratory at 4 °C. Tumor tissues were then transferred to 6 cm culture dishes, where necrotic tissue was excised using sterile scissors, followed by 2–3 washes with basal culture medium (MB-0818L07). The tissues were minced into approximately 1 mm³ fragments and digested in 5 ml of tumor tissue digestion solution (MB-0818L05L) at 37 °C for 30 min. When single cells or small cell clusters appeared, 5 ml of 2% FBS was added and pipetted to stop digestion. The digested cell suspension was filtered through a 100 μm cell strainer, followed by centrifugation at 300 g, 4 °C for 5 min. The supernatant was carefully aspirated, and the cells were washed 2–3 times. In the presence of red blood cells, 3 ml of red blood cell lysis buffer (MB-0818L08L) was added for erythrocyte lysis. Finally, cells were resuspended in 70% Matrigel (abs9490, Absin), seeded into 2–4 wells of a 24-well plate. After polymerization of Matrigel, each well was supplemented with 500 µL of complete human colon cancer medium (MA-0807T001LP) and the cell culture plates were maintained in the incubator for continuous culture.

### Reverse transcription quantitative real-time PCR (RT-qPCR) and mRNA sequencing

Total RNA was extracted using TRNzol Universal Reagent (TIANGEN, DP424), followed by reverse transcription using the PrimeScript™ RT reagent Kit (Takara, RR037Q). RT-qPCR was conducted using Premix Ex Taq™ (Probe qPCR) (Takara, RR39LR). All experiments were independently performed in triplicate. The results are presented as relative gene expression levels normalized to ACTB. The primer sequences of the human genes related to this study are listed in Table. S1. mRNA libraries for sequencing were prepared using the NEB Next Ultra Directional RNA Library Prep Kit for Illumina, following the manufacturer’s protocol. Expression levels of each gene in each sample were quantified using StringTie (v1.3.4) as FPKMs.

### RNA interference

The siRNAs were transfected into cells using GP-transfect-Mate (GenePharma, Shanghai, China) in serum-free medium according to the manufacturer’s instructions. The sequences of the siRNAs used were as follows:

si-NC: 5ʹ-UUCUCCGAACGUGUCACGUTT-3ʹ;

si-SLC9A2#1: 5ʹ- GCUCCAGAACCUGCUCUUUTT-3ʹ;

si-SLC9A2#2: 5ʹ- GCUGCCAUUGUUGUCAUAUTT-3ʹ;

### Establishment of CRC cell lines with high metastatic ability

LoVo and HCT116 cells were initially subjected to transwell assays to evaluate their migration through 8 μm pore membranes. The cells that migrated through the membranes were expanded in culture, and this transwell screening was repeated three times. The resulting in vitro-selected cells were then utilized to establish a liver metastasis model via spleen injection in Nonobese diabetic-SCID (NOD-SCID) mice. Tumor cells were subsequently isolated from liver metastases and expanded in culture, with the in vivo selection process repeated three times. Ultimately, high liver metastatic cell lines were derived and designated as LoVo-Hm or HCT116-Hm.

### Mouse models of liver or peritoneal metastasis models

Female BALB/C nude mice or NOD-SCID mice weighing 15 g were maintained under SPF conditions in accordance with the Institutional Animal Care and Use Committee guidelines of the Beijing Cancer Hospital (Ethics Approval Number: EAEC-2023-13). CRC cells were cultured until reaching approximately 80–90% confluence in 10-cm dishes and subsequently detached using trypsin-EDTA solution. For liver metastasis models, three million CRC cells were injected into the spleen to induce liver metastasis. For peritoneal metastasis models, three million CRC cells were injected into the peritoneal cavity to induce peritoneal metastasis. Tumor growth and metastasis were monitored by injecting luciferin (intraperitoneal, 4 mg/mouse) and imaging with an In Vivo Imaging System twice weekly. Metastatic tumor nodes were quantified at the experimental endpoint following euthanasia of the mice.

### In vivo matrigel plug assay

A total of 500 µL of a mixture containing 250 µL Matrigel Matrix (354248, Corning) and 250 µL CM containing (4 × 10^6^) HUVEC cells was subcutaneously injected into the dorsal flank of nude mice (3 mice per group), forming a firm plug. After five days, the mice were euthanized, and the Matrigel plugs along with surrounding granulation tissue were excised. The Matrigel plugs were fixed in formaldehyde, embedded in paraffin blocks, and sectioned into 4 μm slides for hematoxylin and eosin (H&E) staining and CD31 immunostaining analysis.

### Cell-Derived xenograft and experiments

To establish an MC38 cell line with acquired resistance to immunotherapy, subcutaneous tumor models were developed using wild-type MC38 cells in C57BL/6 mice. When tumor volume reached 50 mm³, each mouse received intraperitoneal injections of 2 mg/kg anti-PD-1 antibody twice weekly. After three weeks, the largest tumors were selected for passaging, performed a total of five times. The dosage of anti-PD-1 antibody was incrementally increased during each passage to 4 mg/kg, 6 mg/kg, 8 mg/kg, and 10 mg/kg. The resulting MC38 tumors, exhibiting resistance to immunotherapy, were designated as MC38-R.

Subcutaneous tumor models were established in C57BL/6 mice using MC38-R cells. MC38-R tumor fragments were excised, cut into small pieces, and implanted subcutaneously in the right flank of the mice. Tumors were measured with calipers, and tumor volume (mm³) was calculated using the formula: tumor volume = (long axis) × (short axis)² × 1/2. Treatments commenced when tumors reached 100 mm³. Mice were randomly assigned to groups based on experimental requirements, with each group receiving either intraperitoneal injections of anti-PD-1 (10 mg/kg, twice weekly), intraperitoneal injections of bevacizumab (15 mg/kg, twice weekly), or oral administration of Ruxolitinib (30 mg/kg, once daily).

### H&E staining and immunohistochemistry (IHC)

The FFPE (Formalin-fixedparaffin-embedded) sections were initially heated at 65 °C for 1 h. Deparaffinization was then carried out with xylene, followed by rehydration through ethanol gradients. Sections were stained with hematoxylin for 5 min, differentiated in 1% hydrochloric acid alcohol, and rinsed thoroughly in tap water until achieving optimal blue coloration. Counterstaining was performed with eosin. Finally, sections were dehydrated in ethanol, cleared with xylene, mounted with a neutral resin, and examined under a microscope for imaging. For IHC analysis, paraffin-embedded samples were baked, deparaffinized, and rehydrated prior to antigen retrieval and blocking of nonspecific antibody binding. Sections were then incubated with specific primary antibodies overnight at 4 °C. After three 5-minute washes with PBS, sections were treated with secondary antibodies for 30 min at room temperature. Following additional washing steps, visualization was achieved using an enzyme substrate, followed by counterstaining with hematoxylin.

For quantification, ImageJ software was employed to analyze IHC and Immunofluorescence (IF) images, allowing for the measurement of average intensity of specific proteins. For each section, three non-overlapping visual fields were randomly selected, and protein expression intensity was assessed. The mean intensity of these fields was calculated for statistical analyses, with the control group’s mean normalized to 1 for graphical representation. All staining, imaging, and quantification procedures were conducted in a blinded manner to maintain the integrity of sample identity and phenotype.

### Public data source and corresponding processing

For bulk data analysis, we utilized publicly available data sources integrated within the BEST database [20], including The Cancer Genome Atlas (TCGA) for CRC, encompassing both colon adenocarcinoma (COAD) and rectum adenocarcinoma (READ), as well as datasets from the Gene Expression Omnibus (GEO) [21].

Single-cell sequencing datasets of CRC cancer were analyzed by Tumor Immune Single-cell Hub (TISCH) [22]. Cancer Single-cell State Atlas (CancerSEA) online database was employed to investigate the function of SLC9A2 in 14 different states in CRC at single-cell resolution, encompassing angiogenesis, apoptosis, invasion, Epithelial-mesenchymal transition (EMT), differentiation, proliferation, DNA damage, metastasis, hypoxia, inflammation, cell cycle, DNA repair, stemness, and quiescence [23]. Other Single- cell data of CRC patients were collected from GEO database (GSE178341, GSE236581). Dimensionality reduction, clustering and cell type annotation of Single-cell RNA sequencing (scRNA-Seq) data are described in the Supplementary Information on Materials and Methods using the “Seurat” R package [24].

### Statistical analysis

All bar graphs were analyzed using GraphPad Prism version 8. The data presented in this study represent the mean ± SEM from triplicate experiments. Two-tailed Student’s t-tests were used for all comparisons between two variables. The Kaplan-Meier curves were compared using the log-rank test, and Spearman correlation analysis was used to assess correlations between two variables. A two-tailed *P*-value < 0.05 was considered statistically significant.

## Results

### Development of a high-metastatic CRC model and exploration of genes driving metastasis

**Fig. 1:**
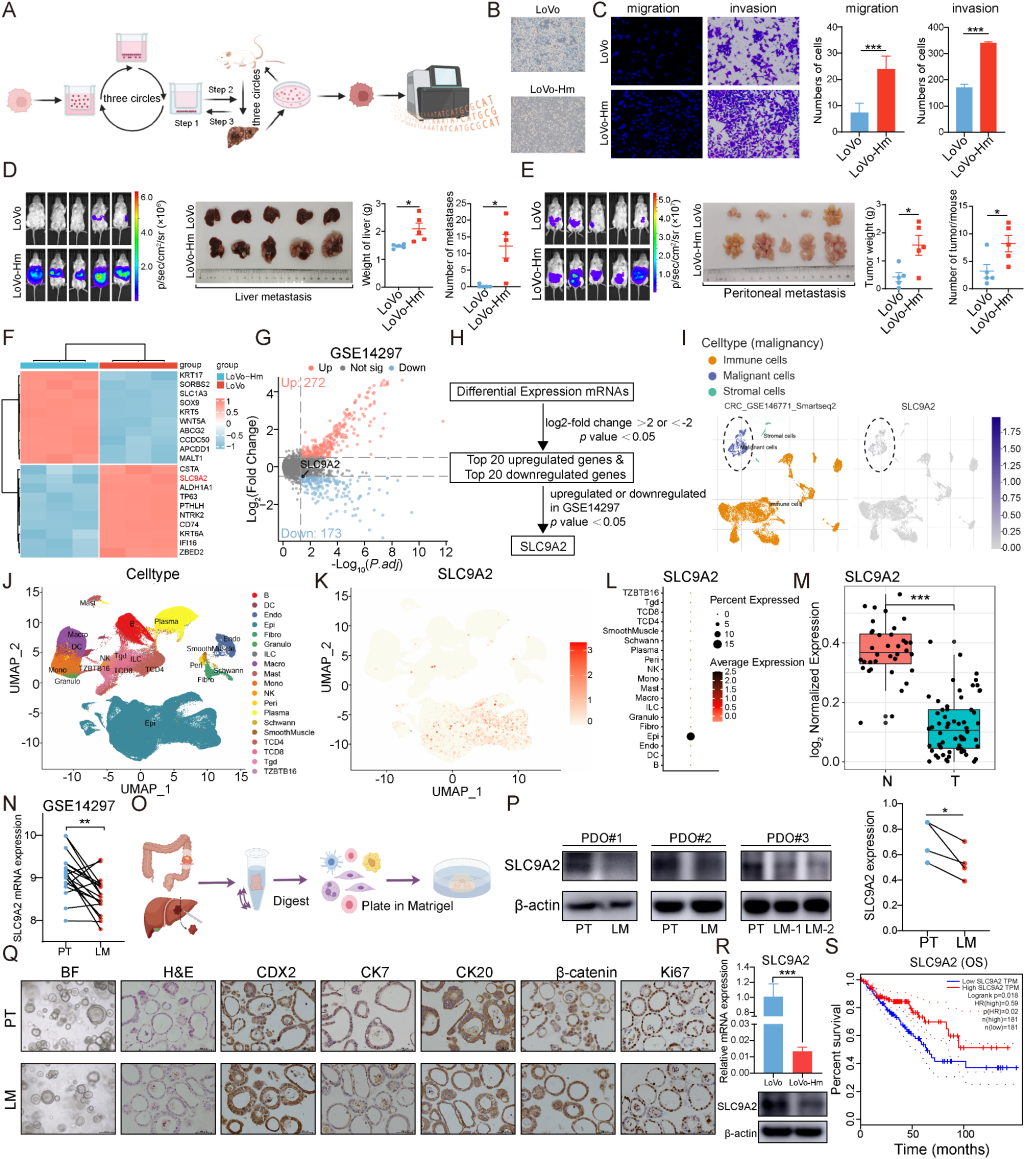
Downregulation of SLC9A2 in hepatic metastases of CRC. (**A**) Schematic diagram showing the construction of a highly liver-metastatic colorectal cancer cell line. LoVo cells were subjected to three rounds of transwell selection, followed by intrasplenic injection in NOD/SCID mice to isolate liver metastases for 2D culture. The highly liver-metastatic cells obtained after three rounds of in *vivo* selection were designated as LoVo-Hm. (**B**) Representative images of LoVo and LoVo-Hm under bright field microscopy. (**C**) LoVo-Hm demonstrated enhanced motility over LoVo following selection, as assessed by migration and invasion assays. Cells were identified in representative images using DAPI for migration and crystal violet for invasion. (**D**) Mouse models of hepatic metastasis were established using LoVo and LoVo-Hm. After four weeks, in vivo bioluminescence imaging was performed, followed by euthanasia to dissect the liver and quantify metastatic lesions and liver weight (n = 5/group). (**E**) Mouse models of peritoneal metastasis were established using LoVo and LoVo-Hm. After four weeks, in vivo bioluminescence imaging was performed, followed by quantification of peritoneal metastatic lesions number and weight (n = 5/group). (**F**) Heatmap analysis of differentially expressed genes between LoVo and LoVo-Hm. (**G-H**) The GSE14297 dataset from the GEO database was selected, and a volcano plot was generated to illustrate differentially expressed genes (**G**) between primary CRC and liver metastases. The top 20 differentially expressed genes (log_2_-fold change > 2 or < -2, *P* < 0.05) were identified, and comparison with the RNA-seq data in Figure F revealed the shared downregulated gene SLC9A2 (**H**). (**I**) Uniform Manifold Approximation and Projection (UMAP) plots of the GSE146771 single-cell sequencing dataset reveals the expression of SLC9A2 in different cell types. (**J-M**) UMAP and dot plots from GSE178341 database illustrate SLC9A2 expression across various cell types, the box plot shows SLC9A2 expression in normal intestinal mucosa (N) versus colorectal cancer (T). (**N**) SLC9A2 expression in paired samples of primary CRC and liver metastases from the GSE14297 database. (**O**) Workflow diagram for the culture of patient-derived organoids from primary CRC and liver metastases. (**P**) Western blot analysis of SLC9A2 expression in PDOs from paired primary CRC and liver metastases (left), with grayscale intensity quantification (right). (**Q**) Representative images of immunohistochemical staining for CDX2, CK7, CK20, β-catenin, and Ki67 in PDOs from the primary CRC tumor and liver metastases. (**R**) Assessment of SLC9A2 levels in LoVo and LoVo-Hm cells was conducted using Western blot and RT-qPCR. (**S**) The association between SLC9A2 expression and OS in TCGA-CRC cohort. Data in bar graphs indicate mean ± SEM. *P < 0.05, **P < 0.01, ***P < 0.001. Student’s t test (**C**, **D**, **E**), paired Student’ s t test (**N**, **P**), log-rank test (**S**)

Tumor heterogeneity manifests in various aspects of cancer, including tumor proliferation, metastasis, and drug resistance. To investigate these characteristics, we selected two cell lines: HCT116, derived from primary CRC, and LoVo, originating from lymph node metastasis, as our primary cells for metastasis and invasion pressure screening. By applying continuous selective evolutionary pressure to both HCT116 and LoVo cells in vivo and in vitro, we successfully established high-metastatic CRC cell lines, HCT116-Hm and LoVo-Hm (Fig. 1A). Compared to their parental cells, the pressure-selected variants exhibited significantly enhanced migratory and invasive capabilities (Fig. 1B-C). Additionally, in vivo assays using peritoneal dissemination and spleen-liver metastasis models further validated that our screening system effectively generates high-metastatic cell lines (Fig. 1D-E). To evaluate the expression profile changes in the pressure-selected cells, we performed transcriptomic sequencing on both LoVo and the pressure-selected derivative, LoVo-Hm (Fig. 1F). Concurrently, we analyzed the GSE14297 dataset, which encompasses sequencing data from primary colorectal cancers and their corresponding liver metastases (Fig. 1G). Initially, we filtered genes with an FPKM value greater than 0. Next, we identified differentially expressed genes (|log_2_FC| > 2, FDR < 0.05) between high-metastatic cells and their parental counterparts, selecting the top 20 upregulated and downregulated genes. We then analyzed differentially expressed genes in primary colorectal tumors compared to paired liver metastases using the GSE14297 dataset. The intersection of these genes with our top candidates revealed SLC9A2 as the only overlapping gene, which we subsequently focused on in our study (Fig. 1H). Notably, SLC9A2 expression was significantly decreased in LoVo-Hm cells and was also found to be markedly lower in liver metastases compared to primary CRC tissues.

To further elucidate whether SLC9A2 is exclusively expressed in tumor cells, we analyzed the GSE146771 single-cell dataset. Our findings revealed that SLC9A2 is expressed solely in malignant epithelial cells, with no detectable expression in stromal or immune cells (Fig. 1I). Additionally, we employed another CRC single-cell dataset, GSE178341, to assess the expression distribution of SLC9A2, confirming that it is limited to the epithelial component of tumors (Fig. 1J-M). Re-examination of SLC9A2 expression in the GSE14297 dataset demonstrated that expression levels in primary tumors (PT) are significantly lower than those in liver metastases (LM) (Fig. 1N).

Following the establishment of paired organoids from primary CRC and liver metastases (Fig. 1O), we conducted H&E staining. The resulting organoids exhibited clear characteristics of tumor cells, including abnormal nuclear-to-cytoplasmic ratios. Pathological analyses confirmed that these organoids displayed expression patterns consistent with CRC (Fig. 1Q). To quantify changes in SLC9A2 protein levels between primary tumors and liver metastases, we extracted proteins from organoids derived from three patients, revealing that SLC9A2 expression in PDOs from liver metastases was significantly lower than in primary CRC (Fig. 1P).

Moreover, we assessed the mRNA and protein levels of SLC9A2 in LoVo and LoVo-Hm cells, discovering a marked downregulation of SLC9A2 in LoVo-Hm cells compared to LoVo cells (Fig. 1R). Finally, Kaplan-Meier analysis indicated that SLC9A2 mRNA levels correlate with overall survival in CRC patients within the TCGA dataset (Fig. 1S).

### Low SLC9A2 expression is associated with advanced TNM staging and poor prognosis of CRC patients

**Fig. 2:**
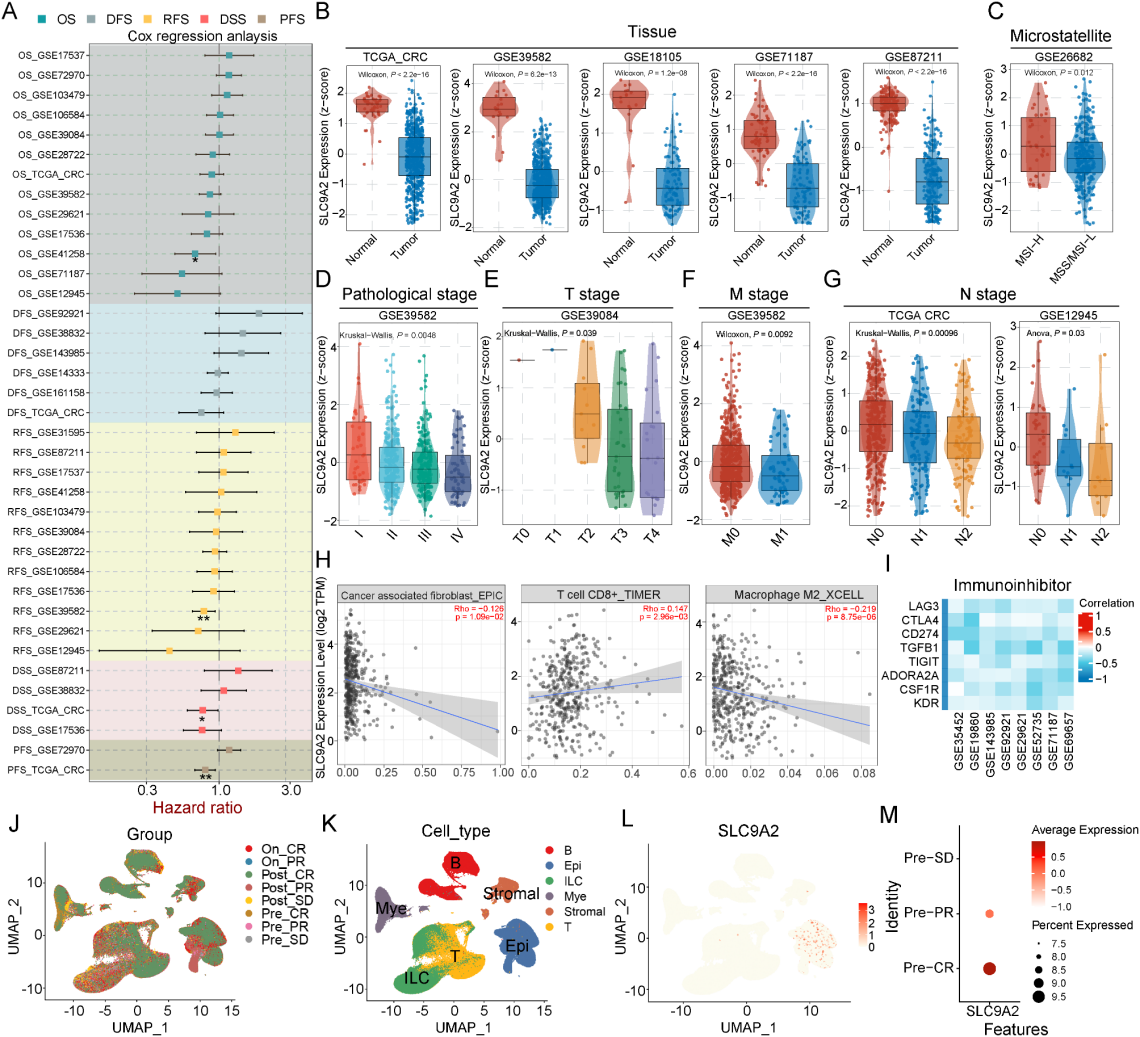
Downregulation of SLC9A2 is associated with adverse clinical features and correlates with an immunosuppressive microenvironment. (**A**) Forest map showing the results of Cox regression analysis on the average survival rate of SLC9A2 in multiple datasets containing survival information. OS for Overall survival; DFS for disease-free survival; RFS for recurrence-free survival; DSS for disease-specific survival and PFS for progression-free survival. (**B**-**G**) Correlation between SLC9A2 and clinical parameters in public databases, including tissue (**B**), microsatellite status (**C**), pathological stage (**D**), T stage (**E**), M stage (**F**), N stage (**G**). T: primary tumor stage; N: regional lymph node stage; M: distant metastasis. (**H**) Correlation analysis between SLC9A2 expression and infiltrating immune cells by TIMER database. (**I**) Analysis of the correlation between SLC9A2 and immunosuppressive molecules using the GEO database. (**J**) UMAP plot of all single cells colored according to the tissue group. (**K**) UMAP plot of all single cells colored according to major cell lineages. (**L**) UMAP plot showed that only epithelial cells express SLC9A2. (**M**) Dot plot of the expression alterations of SLC9A2 in baseline samples from different groups

SLC9A2 expression is significantly downregulated in metastatic sites and is closely associated with poor prognosis in CRC patients. To further explore its relationship with clinical characteristics, we analyzed public datasets from GEO and TCGA. The results indicate that, in a statistically significant manner, SLC9A2 expression levels serve as a favorable prognostic factor for CRC patients, including overall survival (OS), recurrence-free survival (RFS), disease-specific survival (DSS), and progression-free survival (PFS) (Fig. 2A). Subsequently, we evaluated SLC9A2 mRNA expression in CRC specimens using TCGA and GEO databases. In both the TCGA dataset and three GEO datasets, the mRNA expression level of SLC9A2 in CRC tissues was significantly lower than that in adjacent tissues (Fig. 2B). Additionally, SLC9A2 expression was found to be higher in microsatellite instability-high (MSI-H) CRC specimens compared to microsatellite stable (MSS) specimens (Fig. 2C). Notably, a significant decrease in SLC9A2 transcripts was observed in patients with advanced TNM staging of CRC (Fig. 2D). Further results showed that SLC9A2 expression levels progressively declined with the advancement of T/N/M pathological staging (Fig. 2E-G).

Moreover, we utilized the Timer database to analyze the relationship between SLC9A2 expression levels and immune cell infiltration in CRC. The results revealed a negative correlation between SLC9A2 expression and tumor-associated fibroblasts as well as M2 macrophages, while a positive correlation was found with CD8^+^ T cells (Fig. 2H). To assess the impact of SLC9A2 on CRC immunotherapy, we conducted a correlation analysis between SLC9A2 and various immune checkpoint molecules (LAG3, CTLA4, CD274, TGFB1, TIGIT, ADORA2A, CSF1R, KDR) and found that SLC9A2 expression levels were negatively correlated with all eight molecules (Fig. 2I). Therefore, SLC9A2 may play a crucial role in enhancing the efficacy of immunotherapy. To further validate the relationship between SLC9A2 and immunotherapy, we analyzed the CRC immunotherapy single-cell dataset GSE178341. The results indicated that patients with high SLC9A2 expression in baseline tissue specimens exhibited favorable responses to immunotherapy, whereas those with low SLC9A2 expression levels showed no response to treatment (Fig. 2J-M).

### SLC9A2 inhibits malignant progression of CRC cells in vitro

**Fig. 3:**
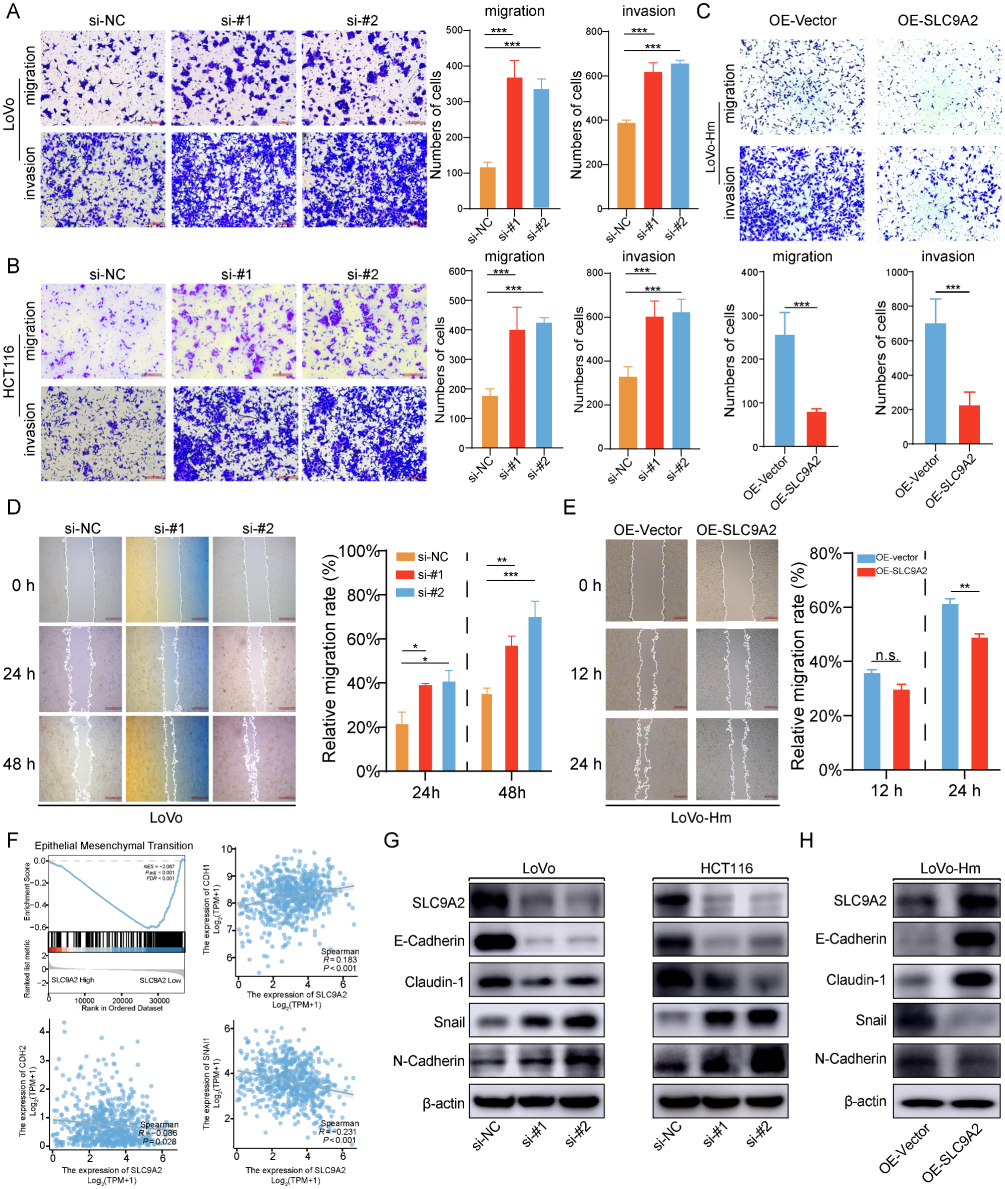
SLC9A2 inhibits the migration and invasion of CRC cells and reverses the EMT process. (**A-B**) Migration and invasion assays were conducted on LoVo (**A**) and HCT116 (**B**) cells transfected with either siRNA-NC or siRNA-SLC9A2. (**C**) Migration and invasion assays were performed on LoVo-Hm cells overexpressing either Vector or SLC9A2. (**D**) The wound healing capacity of LoVo cells transfected with either siRNA-NC or siRNA-SLC9A2 was evaluated. (**E**) The wound healing capacity of LoVo cells overexpressing either Vector or SLC9A2 was assessed. (**F**) GSEA analysis was performed to explore the relationship between SLC9A2 expression and pathway enrichment. Spearman’ s correlation analysis was utilized to assess the correlation between the expression levels of CDH1, CDH2, SNAIL, and SLC9A2, using data from TCGA (**G**) Western blot was used to measure the expression levels of SLC9A2, E-Cadherin, N-Cadherin, Claudin-1, Snail in LoVo (left) and HCT116 (right) cells transfected with either siRNA-NC or siRNA-SLC9A2. (**H**) Western blot was used to measure the expression levels of SLC9A2, E-Cadherin, N-Cadherin, Claudin-1, Snail in LoVo-Hm cells overexpressing either Vector or SLC9A2. Data in bar graphs indicate mean ± SEM. *P < 0.05, **P < 0.01, ***P < 0.001, n.s., not significant. Multi-group analysis of variance (**A**, **B**, **D**), Student’s t test (**C**, **E**)

To further confirm the tumor-suppressive role of SLC9A2 in CRC, we investigated its biological function by transfecting colorectal cancer cells with SLC9A2 overexpression plasmids and siRNA. The efficiency of gain- and loss-of-function was validated by RT-qPCR (Figure S1). Given that SLC9A2 was identified through a metastasis model, we focused on its impact on tumor cell migration and invasion. As shown in the Transwell assays, SLC9A2 knockdown significantly reduced the number of cells migrating to the lower chamber. Additionally, the invasive capacity of CRC cells through Matrigel was diminished following SLC9A2 suppression (Fig. 3A-B). To further elucidate the role of SLC9A2 in CRC cells, we restored SLC9A2 expression in LoVo-Hm cells. Induction of SLC9A2 expression in LoVo-Hm resulted in an decreased number of CRC cells migrating through the Transwell membrane and invading the Matrigel (Fig. 3C). Moreover, in a wound healing assay, migration distance was enhanced in siRNA-transfected LoVo cells (Fig. 3D), while SLC9A2 overexpression in LoVo-Hm cells led to a reduced migration distance (Fig. 3E). These results indicate that SLC9A2 inhibits the migration and invasion of CRC cells.

EMT is a hallmark of cancer that is essential for tumor metastasis and is closely associated with cell migration and invasion. To elucidate the impact of SLC9A2 on the EMT process in CRC cells, we performed Gene Set Enrichment Analysis (GSEA). The results indicated that the EMT process was significantly enhanced in populations with low SLC9A2 expression (Fig. 3F). Furthermore, SLC9A2 expression levels showed a positive correlation with the epithelial marker CDH1 and an inverse correlation with the mesenchymal markers CDH2 and SNAIL1 (Fig. 3F). These findings suggest that SLC9A2 inhibits the occurrence of EMT in CRC.

Additionally, we assessed the effect of SLC9A2 on EMT markers. Western blot analysis revealed that SLC9A2 knockdown upregulated mesenchymal markers (N-cadherin and Snail) and increased the expression of epithelial markers (E-cadherin and Claudin-1). Conversely, SLC9A2 overexpression resulted in decreased levels of N-cadherin and Snail, while E-cadherin and Claudin-1 expression was elevated (Fig. 3G-H). Collectively, our results indicate that SLC9A2 suppresses the migration and invasion capabilities of CRC cells.

### SLC9A2 inhibits liver metastasis of CRC in vivo

**Fig. 4:**
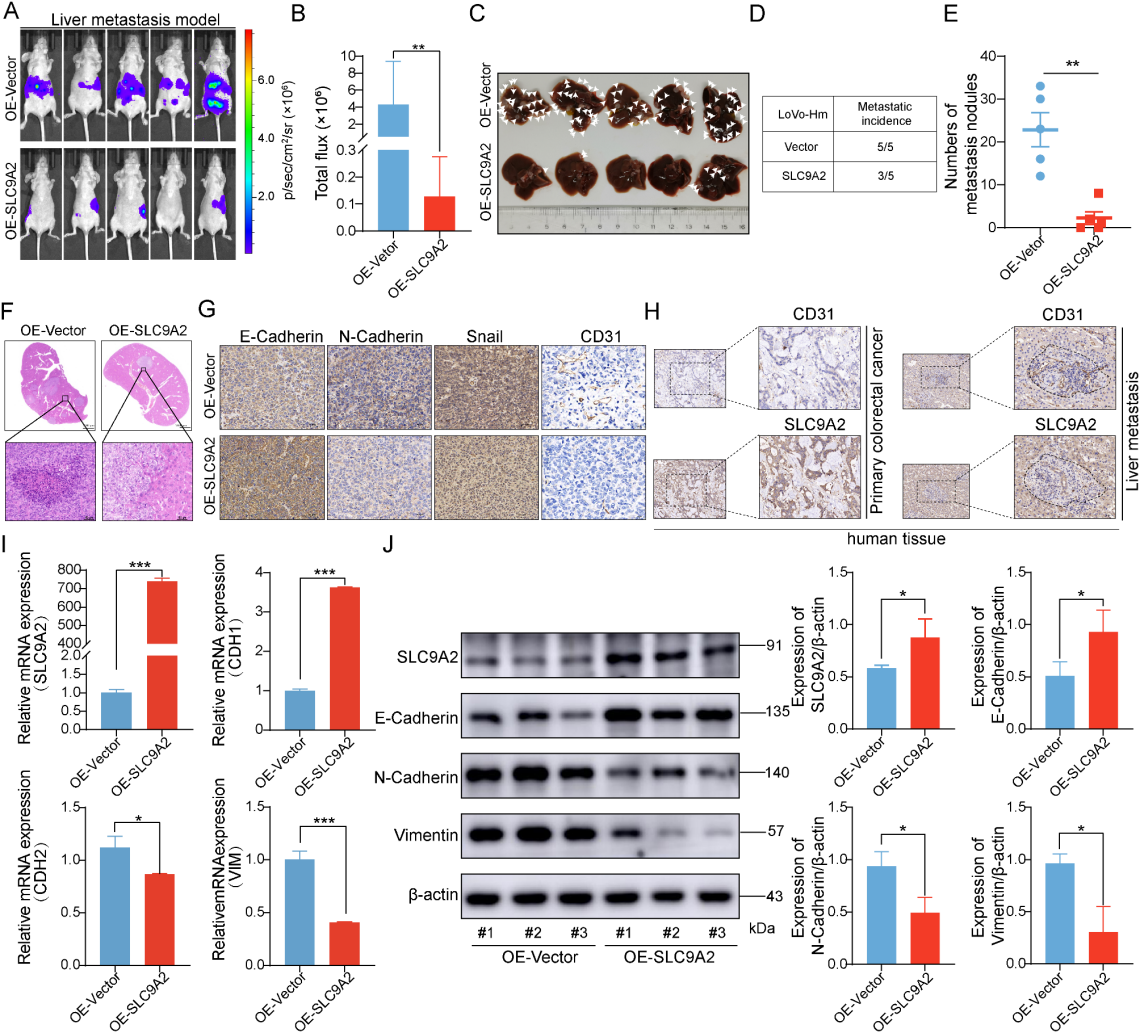
SLC9A2 inhibits the hepatic metastasis of colorectal cancer in vivo. (**A-B**) BALB/c nude mice models of hepatic metastasis were established using LoVo-Hm cells overexpressing either Vector or SLC9A2. After four weeks, in vivo bioluminescence imaging was performed (*n* = 5/group). (**C-E**) BALB/c nude mice were euthanized to dissect the liver and quantify metastatic lesions. (**F**) Representative images of H&E staining of BALB/c nude mice livers are presented. (**G**) IHC analysis was conducted to assess the expression of E-Cadherin, N-Cadherin, Snail, and CD31 in the liver metastatic lesions of BALB/c nude mice. (**H**) IHC analysis was performed to evaluate the expression of CD31 and SLC9A2 in the primary lesions and liver metastases of CRC patients. (**I-J**) RT-qPCR (**I**) and Western blot (**J**) analyses were performed to measure the levels of SLC9A2, E-Cadherin, N-Cadherin, and Vimentin in the liver metastatic lesions of BALB/c nude mice. Data in bar graphs indicate mean ± SEM, and data were analyzed using Student’s t tests. *P < 0.05, **P < 0.01, ***P < 0.001

To assess the in vivo function of SLC9A2, we investigated its impact on tumor cell migration and invasion using a CRC spleen-liver metastasis model. We observed a significant reduction in the number of liver metastatic nodules in the SLC9A2 overexpression group compared to the control group (Fig. 4A-E). H&E staining of liver sections revealed markedly fewer metastatic foci in the SLC9A2 overexpression group (Fig. 4F). IHC results indicated that SLC9A2 overexpression significantly increased the expression of the epithelial marker E-cadherin while decreasing the expression of mesenchymal markers N-cadherin and Snail. Additionally, we assessed the expression level of CD31, an angiogenesis marker, in liver metastatic tissues following SLC9A2 overexpression. The results showed that CD31 expression was significantly reduced in tumor tissues, suggesting that SLC9A2 may be involved in the angiogenic process of tumors (Fig. 4G). Furthermore, we performed IHC staining on primary CRC lesions and corresponding liver metastases from the same patient. The results indicated that SLC9A2 expression levels were higher in primary tumors compared to liver metastases, while CD31 exhibited higher expression in liver metastases than in primary lesions. This observation further supports a reciprocal relationship between SLC9A2 and CD31, suggesting a role for SLC9A2 in tumor angiogenesis (Fig. 4H).

In addition, we collected specimens from liver metastatic lesions for RT-qPCR and Western blot analyses. As shown in Fig. 4I-J, both RNA and protein levels confirmed that SLC9A2 overexpression upregulated E-cadherin and downregulated N-cadherin and Vimentin expression. Collectively, these results indicate that SLC9A2 may inhibit angiogenesis and metastasis in colorectal cancer in vivo.

### SLC9A2 inhibits the JAK/STAT3 signaling pathway to suppress metastasis of CRC

**Fig. 5:**
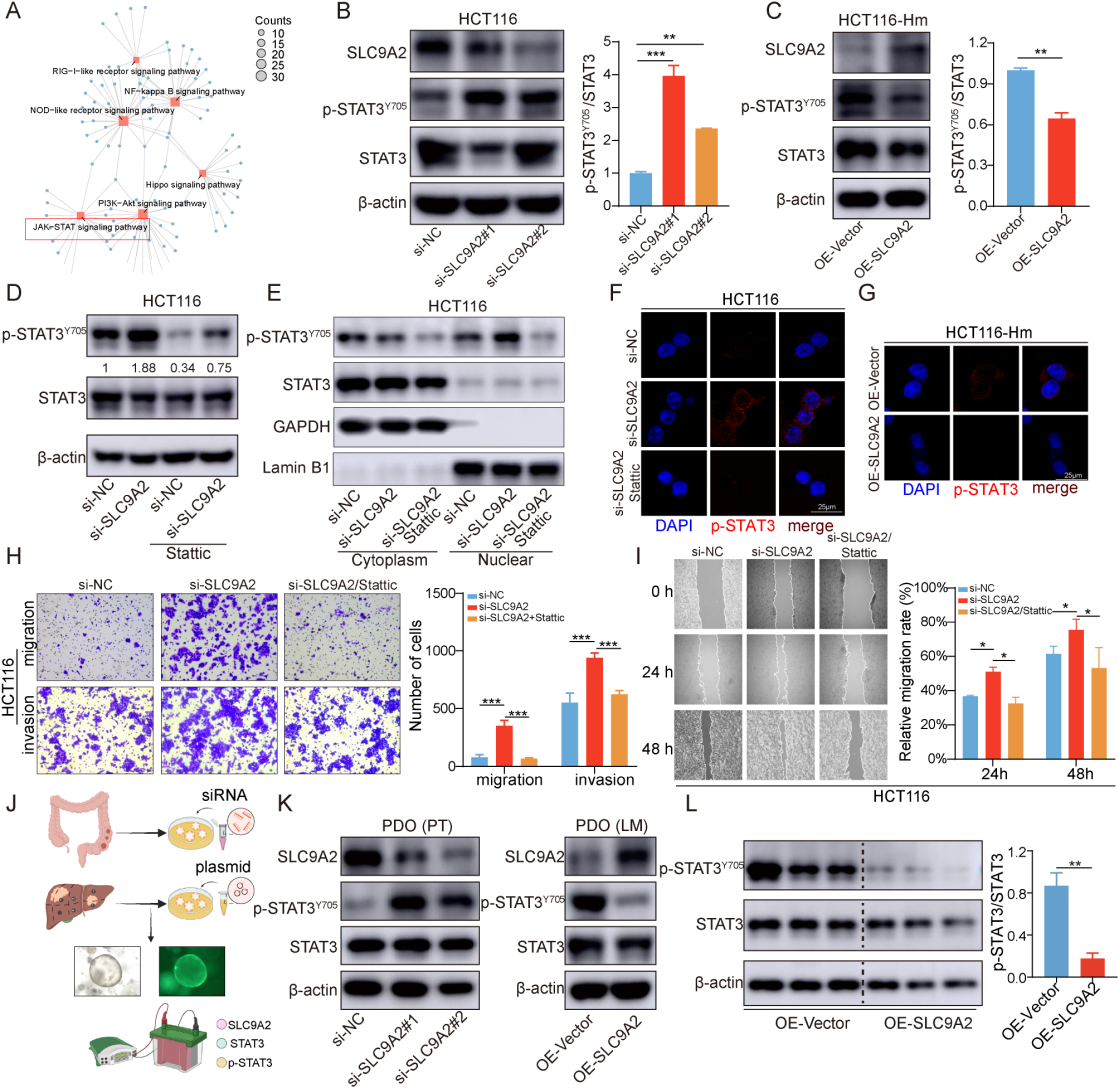
SLC9A2 inhibits the migration and invasion of CRC cells by suppressing the STAT3 signaling pathway. (**A**) Pathway enrichment analysis was conducted to compare the SLC9A2-overexpressing CRC cells with the control group. (**B**) Western blot analysis was performed to measure the levels of SLC9A2, STAT3 and p-STAT3^Y705^ in HCT116 cells transfected with either siRNA-NC or siRNA-SLC9A2. (**C**) Western blot analysis was performed to measure the levels of SLC9A2, STAT3 and p-STAT3^Y705^ in HCT116-Hm cells overexpressing either Vector or SLC9A2. (**D**) HCT116 cells were transfected with either siRNA-NC or siRNA-SLC9A2, followed by treatment with or without 5 µM Stattic for 24 h. After 48 h, Western blot analysis was performed to evaluate the levels of STAT3 and p-STAT3^Y705^. (**E-F**) HCT116 cells were transfected with either siRNA-NC or siRNA-SLC9A2, and the siRNA-SLC9A2 group was subsequently treated with 5 µM Stattic for 24 h. Following a 48-hour incubation, nuclear-cytoplasmic separation was performed, and Western blot analysis was conducted to evaluate the levels of STAT3 and p-STAT3^Y705^ (**E**). Immunofluorescence was used to assess the nuclear localization of p- STAT3^Y705^ (**F**). (**G**) HCT116-Hm cells were transfected with either vector or SLC9A2. After 24 h, immunofluorescence was used to examine the nuclear localization of p-STAT3^Y705^. (**H**) Migration and invasion assays were conducted on HCT116 cells transfected with either siRNA-NC or siRNA-SLC9A2, and the siRNA-SLC9A2 group was subsequently treated with 5 µM Stattic for 24 h. (**I**) Wound healing assay was conducted on HCT116 cells transfected with either siRNA-NC or siRNA-SLC9A2, and the siRNA-SLC9A2 group was subsequently treated with 5 µM Stattic for 24 h. (**J**) Schematic diagram illustrating SLC9A2 knockdown and overexpression in PDOs from primary CRC and liver metastases to evaluate STAT3 activation. (**K**) Western blot analysis of p-STAT3^Y705^ levels in PDOs with SLC9A2 knockdown from primary CRC (left) and overexpression from liver metastases (right). (**L**) Western blot analysis of p-STAT3^Y705^ levels in mouse liver metastases as shown in Fig. 4C. Data in bar graphs indicate mean ± SEM. *P < 0.05, **P < 0.01, ***P < 0.001. Multi-group analysis of variance (**B**, **H**, **I**), Student’s t test (**C**, **L**)

Subsequently, we investigated the mechanism by which SLC9A2 mediates the inhibition of CRC metastasis. To identify differentially expressed genes between SLC9A2-overexpression and control cells, we performed RNA sequencing. Kyoto Encyclopedia of Genes and Genomes (KEGG) analysis revealed that the differentially expressed genes were associated with the JAK/STAT signaling pathway (Fig. 5A). Notably, activation of the JAK/STAT3 signaling pathway is involved in multiple aspects of tumorigenesis, progression, invasion, and metastasis. The phosphorylation of STAT3 at Y705 (pSTAT3^Y705^) is a critical step in activating the STAT3 signaling pathway. To validate this, we first knocked down SLC9A2 in HCT116 and LoVo cells and found increased expression of p-STAT3^Y705^ (Fig. 5B, Figure S2A). Conversely, overexpression of SLC9A2 in HCT116-Hm and LoVo-Hm cells significantly downregulated p-STAT3^Y705^ expression (Fig. 5C, Figure S2B). To demonstrate that the downregulation of SLC9A2 primarily promotes the accumulation of p-STAT3^Y705^ and its nuclear translocation leading to transcriptional activation, we employed Stattic to inhibit STAT3 phosphorylation. Stattic treatment abolished the increase in p-STAT3^Y705^ accumulation observed after SLC9A2 knockdown (Fig. 5D, Figure S2C). Nuclear-cytoplasmic fractionation experiments revealed that the level of p-STAT3^Y705^ protein in the nucleus was significantly increased in the si-SLC9A2 group compared to the si-NC group, and this was similarly inhibited by the Stattic inhibitor (Fig. 5E, Figure S2D). Immunofluorescence staining of p-STAT3 further confirmed these results, showing reduced nuclear translocation of p-STAT3 after SLC9A2 overexpression (Fig. 5F-G, Figure S2E-F).

The impact of SLC9A2 knockdown on cell migration and invasion was profound in both HCT116 and LoVo cell lines. However, the promotion of CRC cell migration and invasion by SLC9A2 knockdown was markedly diminished following Stattic treatment (Fig. 5H-I; Figure S2G-H). Additionally, we established organoid models using primary CRC lesions and corresponding liver metastatic lesions from the same patient (Fig. 5J). Knockdown of SLC9A2 in primary lesions led to increased expression of p-STAT3^Y705^ in the organoids derived from liver metastases. Conversely, overexpression of SLC9A2 in the liver metastatic organoids suppressed p-STAT3^Y705^ levels (Fig. 5K). Simultaneously, we performed Western blot analysis on liver metastatic lesions obtained from the mouse model in Fig. 4, confirming that overexpression of SLC9A2 in vivo also inhibited p-STAT3^Y705^ levels (Fig. 5L). Collectively, these results underscore that SLC9A2 exerts anti-tumor effects by inhibiting p-STAT3^Y705^ expression.

### SLC9A2 inhibits VEGF-A expression and curbs tumor angiogenesis

**Fig. 6:**
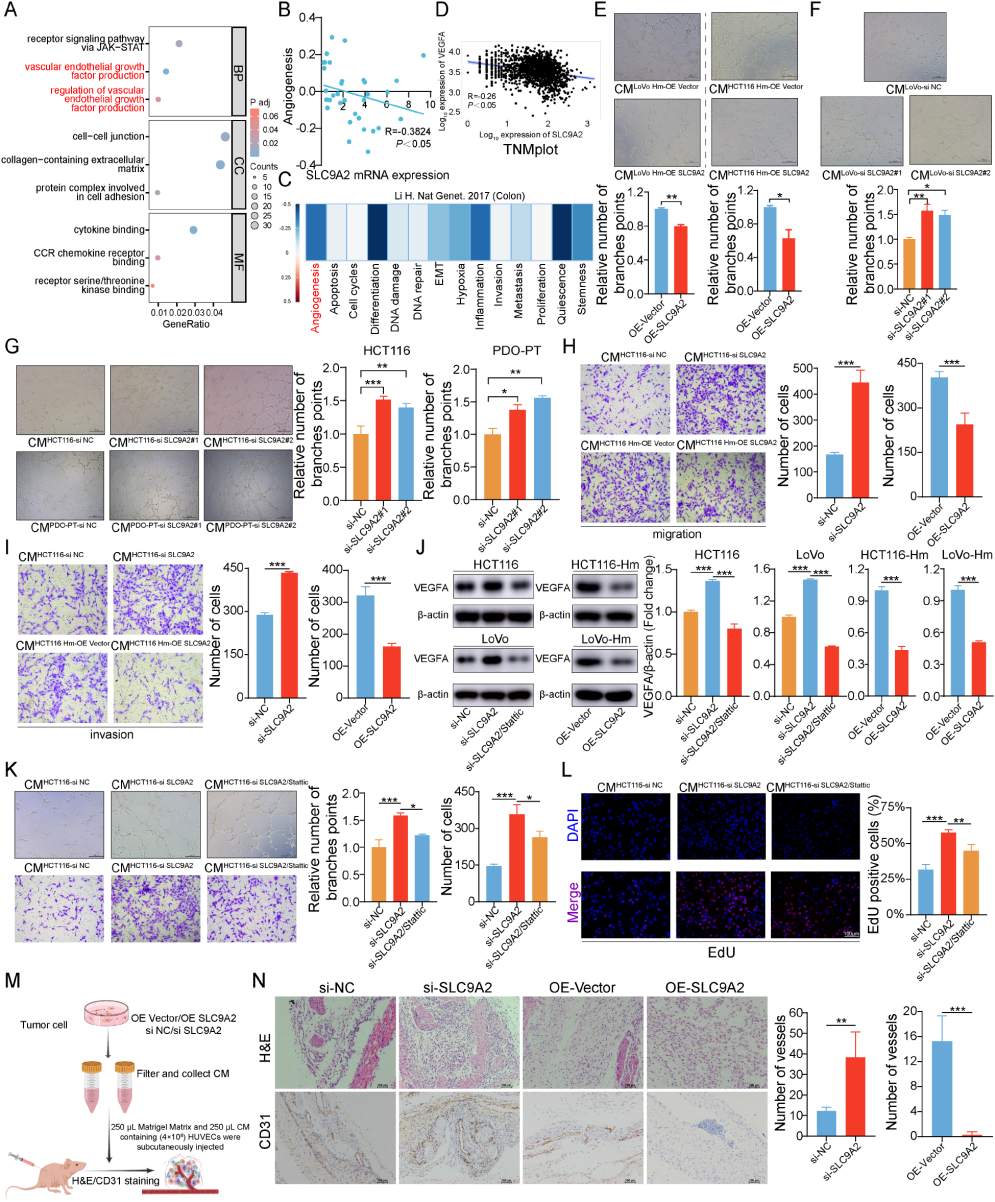
SLC9A2 inhibits VEGFA expression and curbs tumor angiogenesis. (**A**) Gene Ontology analysis was performed to compare SLC9A2-overexpressing CRC cells with the control group. (**B**-**C**) Analysis of the correlation between SLC9A2 expression and angiogenesis pathway in the CancerSEA web server. (**D**) Analysis of the correlation between SLC9A2 expression and angiogenesis pathway genes in TNMplot database. (**E**) CM from LOVO-Hm or HCT116-Hm cells transfected with either an overexpression vector or SLC9A2 were used to stimulate HUVEC cells for tubule formation assays. (**F**) CM from LOVO transfected with either si-NC or si-SLC9A2 were used to stimulate HUVEC cells for tubule formation assays. (**G**) CM from HCT116 or CRC PDOs transfected with either si-NC or si-SLC9A2 were used to stimulate HUVEC cells for tubule formation assays. (**H**) CM from HCT116 cells transfected with either si-NC or si-SLC9A2 (top), and from HCT116-Hm cells transfected with either an overexpression vector or SLC9A2 (bottom), were used to stimulate HUVEC cells in migration assays. (**I**) CM from HCT116 cells transfected with either si-NC or si-SLC9A2 (top), and from HCT116-Hm cells transfected with either an overexpression vector or SLC9A2 (bottom), were used to stimulate HUVEC cells in invasion assays. (**J**) Western blot analysis was conducted to assess the expression levels of VEGFA in LOVO and HCT116 cells transfected with either si-NC or si-SLC9A2, as well as in LOVO-Hm or HCT116-Hm cells transfected with either an overexpression vector or SLC9A2 (left). Grayscale values were quantified (right). (**K**) HCT116 cells were transfected with either siRNA-NC or siRNA-SLC9A2, and the siRNA-SLC9A2 group was treated with 5 µM Stattic for 24 h. CM from the three groups were subsequently collected to stimulate HUVEC cells for tubule formation (top) and migration assay (bottom). (**L**) CHCT116 cells were transfected with either siRNA-NC or siRNA-SLC9A2, and the siRNA-SLC9A2 group was treated with 5 µM Stattic for 24 h. CM from the three groups were subsequently collected to stimulate HUVEC cells for EdU assay. (**M**) Flowchart of the in vivo tubule formation assay. (**N**) CM were collected from HCT116 cells transfected with either si-NC or si-SLC9A2, as well as from HCT116-Hm cells transfected with an overexpression vector or SLC9A2. HUVEC cells were then mixed with the CM and subcutaneously injected into nude mice (*n* = 3/group). After 7 days, the implants were harvested for H&E staining and CD31 IHC (left panel), followed by quantification of the number of tubules formed (right panel). Data in bar graphs indicate mean ± SEM. *P < 0.05, **P < 0.01, ***P < 0.001. Multi-group analysis of variance (**F**, **G**, **J**, **K**, **L**), Student’s t test (**E**, **H**, **I**, **J**, **N**)

Overexpression of SLC9A2 in highly metastatic cells markedly reduces their ability to metastasize to the liver in vivo. Beyond inhibiting tumor cell migration through the suppression of the STAT3 signaling pathway, SLC9A2 may exert additional biological functions that contribute to the inhibition of CRC cell metastasis. Gene Ontology (GO) analysis of differentially expressed genes revealed enrichment not only in the JAK-STAT3 pathway but also in the VEGF signaling pathway, suggesting a potential role of SLC9A2 in tumor angiogenesis (Fig. 6A). It is well established that angiogenesis is a critical facilitator of early cancer metastasis, marking the transition of tumors from a dormant state to a proliferative phase, thereby accelerating tumor growth and promoting metastasis. Thus, the biological processes underlying angiogenesis are closely linked to the migratory and invasive tendencies of CRC cells.

To elucidate the functional status of SLC9A2 in CRC cells at single-cell resolution, we utilized the CancerSEA web server. As depicted in Fig. 6B-C, in the single-cell RNA sequencing dataset of CRC, SLC9A2 expression negatively correlates with the malignant phenotypes of tumors, including a significant inverse relationship with tumor angiogenesis. These findings imply a role for SLC9A2 in the anti-angiogenic processes. During IHC staining, we observed reduced SLC9A2 expression in liver metastatic lesions compared to primary tumors. Furthermore, CD31, a marker of blood vessels, was expressed at higher levels in liver metastases with lower SLC9A2 expression, indicating that SLC9A2 possesses the capability to inhibit angiogenesis (Fig. 4H).

As mounting evidence highlights the vital role of VEGFA in tumor angiogenesis, we subsequently examined the connection between SLC9A2 and VEGFA. Correlation analysis using the TNMplot database revealed a negative correlation between SLC9A2 and VEGFA expression (Fig. 6D). To further clarify the impact of SLC9A2 on tumor angiogenesis, we conducted tube formation assays using Human Umbilical Vein Endothelial Cells (HUVECs). The conditioned media from SLC9A2-overexpressing CRC tumor cells significantly inhibited HUVEC tube formation, whereas silencing SLC9A2 markedly promoted tube formation. Additionally, knockdown of SLC9A2 in CRC-derived organoids stimulated HUVEC cells, further confirming that SLC9A2 knockdown enhances tube formation (Fig. 6E-G).

Given that endothelial cell migration is critical for angiogenesis, we employed transwell assays to assess the effects of SLC9A2 on HUVEC migration. Results indicated that the conditioned media from SLC9A2-overexpressing tumor cells significantly inhibited HUVEC migration and invasion, while media from SLC9A2-knockdown cells promoted these processes (Fig. 6H-I). GO analysis of differentially expressed genes also indicated enrichment in the JAK-STAT3 pathway. Our preliminary experimental data demonstrated that highly metastatic tumor cells activate the STAT3 signaling pathway by downregulating SLC9A2, facilitating tumor cell migration and invasion, leading us to hypothesize that STAT3 signaling might also be involved in the angiogenic pathway. To test this hypothesis, we introduced the STAT3 phosphorylation inhibitor Stattic into SLC9A2-knockdown tumor cells and observed that the upregulation of VEGFA induced by SLC9A2 knockdown was reversed (Fig. 6J). Further tube formation assays, migration assays, and EdU proliferation assays demonstrated that the promotion of HUVEC tube formation, migration, and proliferation resulting from SLC9A2 knockdown could be rescued by Stattic (Fig. 6K-L). Therefore, SLC9A2’s inhibition of tumor angiogenesis is associated with its suppression of the STAT3 signaling pathway.

Consistent angiogenic effects were also observed in the matrigel plug assay conducted in nude mice. H&E and CD31 IHC staining analysis of the paraffin sections of the viscous plugs revealed that a substantial number of blood vessels infiltrated the plugs in the co-culture group of HCT116 si-SLC9A2 cells compared to HCT116 si-NC cells. Conversely, overexpression of SLC9A2 resulted in a significant reduction in angiogenesis in vivo, suggesting that SLC9A2 can significantly inhibit the angiogenic capacity of HUVECs both in vitro and in vivo (Fig. 6M-N).

### Slc9a2 effectively reverses immune resistance in colorectal cancer by inhibiting angiogenesis

As highlighted in Fig. 2, we observed that higher baseline SLC9A2 expression levels in the CRC immunotherapy cohort correlated with improved responses to treatment. Recent studies have underscored the role of anti-angiogenic therapies in enhancing effector T-cell infiltration while promoting vascular normalization, which in turn reduces the recruitment of regulatory T cells and myeloid-derived suppressor cells [25]. Based on these findings, we propose that SLC9A2 inhibits the STAT3 signaling pathway, thereby suppressing metastasis and tumor angiogenesis in CRC. Consequently, we hypothesize that SLC9A2 may synergistically enhance immunotherapy by inhibiting the STAT3 pathway.

**Fig. 7:**
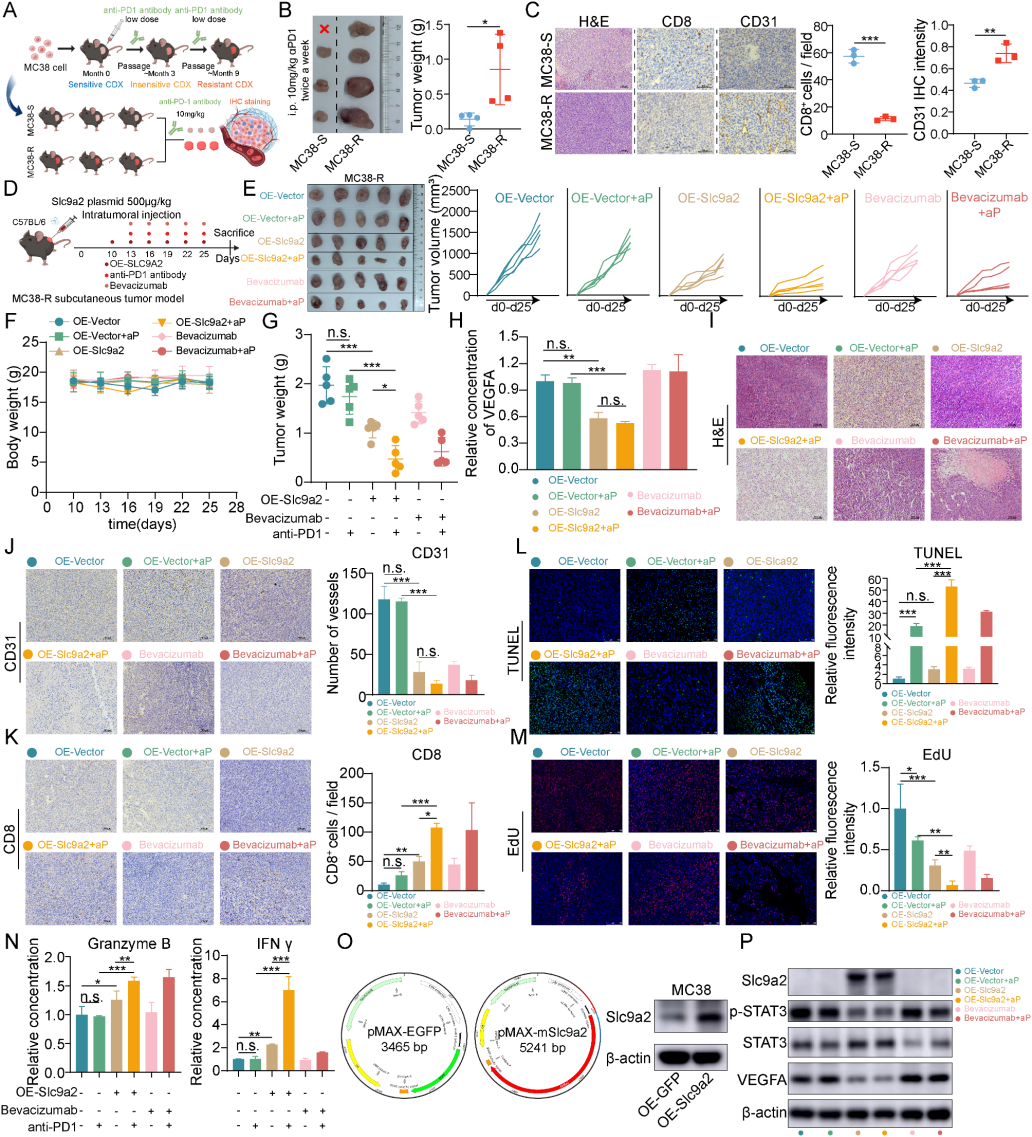
Slc9a2 effectively reverses immune resistance in colorectal cancer by inhibiting angiogenesis. (**A**) Flowchart for the establishment of the MC38-R cell line demonstrating resistance to immunotherapy. MC38 cells that developed acquired resistance to immunotherapy were selected under low-dose PD-1 antibody pressure and designated as MC38-R. (**B**) Subcutaneous tumor models in C57BL/6 mice were constructed using both MC38-S (sensitive) and MC38-R cell lines (*n* = 4/group). Subsequent intraperitoneal injection of PD-1 monoclonal antibody was conducted to evaluate the efficacy of immunotherapy based on tumor weight. (**C**) Three mice from each group depicted in Fig. **7B** were selected for H&E staining, as well as IHC staining for CD8 and CD31, followed by statistical analysis. (**D**) MC38-R cells were employed to establish subcutaneous tumor models in C57BL/6 mice. Upon reaching a tumor volume of 100 mm³, an SLC9A2 overexpression plasmid was intratumorally injected, accompanied by intraperitoneal administration of anti-PD-1 and oral administration of Bevacizumab. (**E-G**) Following four weeks of MC38-R subcutaneous tumor implantation, the mice were sacrificed, and tumors were dissected for photography and weight measurement (**G**). The volume changes of tumor growth were analyzed for each group (**E**), alongside assessments of body weight variations in the mice (**F**). (**H**) VEGFA levels in tumor tissues from each group of mice were quantified using ELISA, with the control group mean normalized to 1 for statistical analysis and graphical representation. (**I-M**) Tumor tissues depicted in Fig. **7E** were embedded in paraffin and subjected to H&E staining (**I**), as well as CD31 (**J**) and CD8 (**K**) immunohistochemistry. Additionally, TUNEL (**L**) and EdU (**M**) staining were performed to assess tumor proliferation and apoptosis. (**N**) Granzyme B (left) and IFNγ (right) levels in tumor tissues from each group of mice were quantified using ELISA, with the control group mean normalized to 1 for statistical analysis and graphical representation. (**O**) Map of the slc9a2 pcDNA plasmid and validation of the transfection efficiency in MC38. (**P**) Western blot analysis was conducted to determine the levels of VEGFA and p-STAT3^Y705^. Data in bar graphs indicate mean ± SEM. *P < 0.05, **P < 0.01, ***P < 0.001, n.s., not significant. Student’s t test (**B**, **C**), multi-group analysis of variance (**G**, **H**, **J**, **K**, **L**, **M**, **N**)

To investigate this, we established MC38-R, a subline that acquires resistance to immunotherapy through long-term, low-dose induction with an anti-PD-1 antibody in MC38 (Fig. 7A-B). IHC analysis of subcutaneous tumors from both MC38 and MC38-R post-immunotherapy revealed significantly reduced CD8^+^ T-cell infiltration in MC38-R tumors, along with a marked increase in vascular density. These results further corroborate the activation of tumor angiogenesis pathways as contributors to the process of immunotherapy resistance (Fig. 7C).

We devised an in-situ overexpression strategy for Slc9a2 in MC38-R xenografts, as previously reported. Initially, we validated the expression of Slc9a2 in MC38 cells via plasmid transfection in vitro (Fig. 7O). Following a 10-day period of subcutaneous tumor implantation, mice received two weeks of intratumoral injections of either the Slc9a2 overexpression plasmid vector plus delivery buffer or the control vector plus delivery buffer, with bevacizumab serving as a positive control. Monotherapy with anti-PD-1 was ineffective in inhibiting tumor growth; however, both the Slc9a2 plasmid and bevacizumab markedly enhanced the anti-tumor efficacy of the anti-PD-1 antibody against MC38-R xenografts (Fig. 7D-G).

Consistent with the observed inhibition of VEGFA expression in tumor cells due to SLC9A2, ELISA results confirmed a reduction in VEGFA levels in the Slc9a2 overexpression group (Fig. 7H). IHC analysis of CD31 in the cell line-derived xenografts (CDX) demonstrated that the number of blood vessels in the OE-Slc9a2 + anti-PD-1 and bevacizumab + anti-PD-1 groups was significantly lower than in other groups (Fig. 7J). Furthermore, analysis of tumor-infiltrating immune cells revealed increased CD8^+^ TILs in both the OE-Slc9a2 + anti-PD-1 and bevacizumab + anti-PD-1 groups (Fig. 7K).

In alignment with the enhanced anti-PD-1 efficacy mediated by Slc9a2 and bevacizumab, the number of terminal deoxynucleotidyl transferase dUTP nick end labeling (TUNEL) apoptotic cells increased in the OE-Slc9a2 + anti-PD-1 and bevacizumab + anti-PD-1 groups compared to all other groups (Fig. 7L), alongside decreased cell proliferation as assessed by EdU staining (Fig. 7M). ELISA experiments validated that OE-Slc9a2 + anti-PD-1 treatment stimulated granzyme B/IFN-γ production, particularly enhancing IFN-γ levels (Fig. 7N). Western blot analysis further demonstrated the efficiency of Slc9a2 overexpression within the tumors, revealing reduced levels of p-STAT3 and VEGFA expression (Fig. 7P). Collectively, these data indicate that Slc9a2 may reduce VEGFA expression through the inhibition of the STAT3 pathway, thus reversing the effects of immunotherapy resistance.

### Ruxolitinib augments the effectiveness of Anti-PD-1 immunotherapy against CRC

Currently, there are no effective drugs for specifically upregulating SLC9A2 expression, and there are no mature targeted therapies for the activation of the STAT3 pathway. Inhibiting the upstream signals of STAT3 represents a promising strategy, as JAK is the most crucial upstream signaling factor for STAT3. Studies have shown that Ruxolitinib, a JAK inhibitor, can synergize with immunotherapy to enhance treatment efficacy in Hodgkin lymphoma and non-small cell lung cancer [26, 27]. Given that Ruxolitinib has been reported to modulate melanoma cell-intrinsic resistance to anti-PD-1 [28], we wondered whether Ruxolitinib could mimic the function of SLC9A2 by inhibiting the STAT3 signaling pathway, thereby reversing immunotherapy resistance in CRC.

**Fig. 8:**
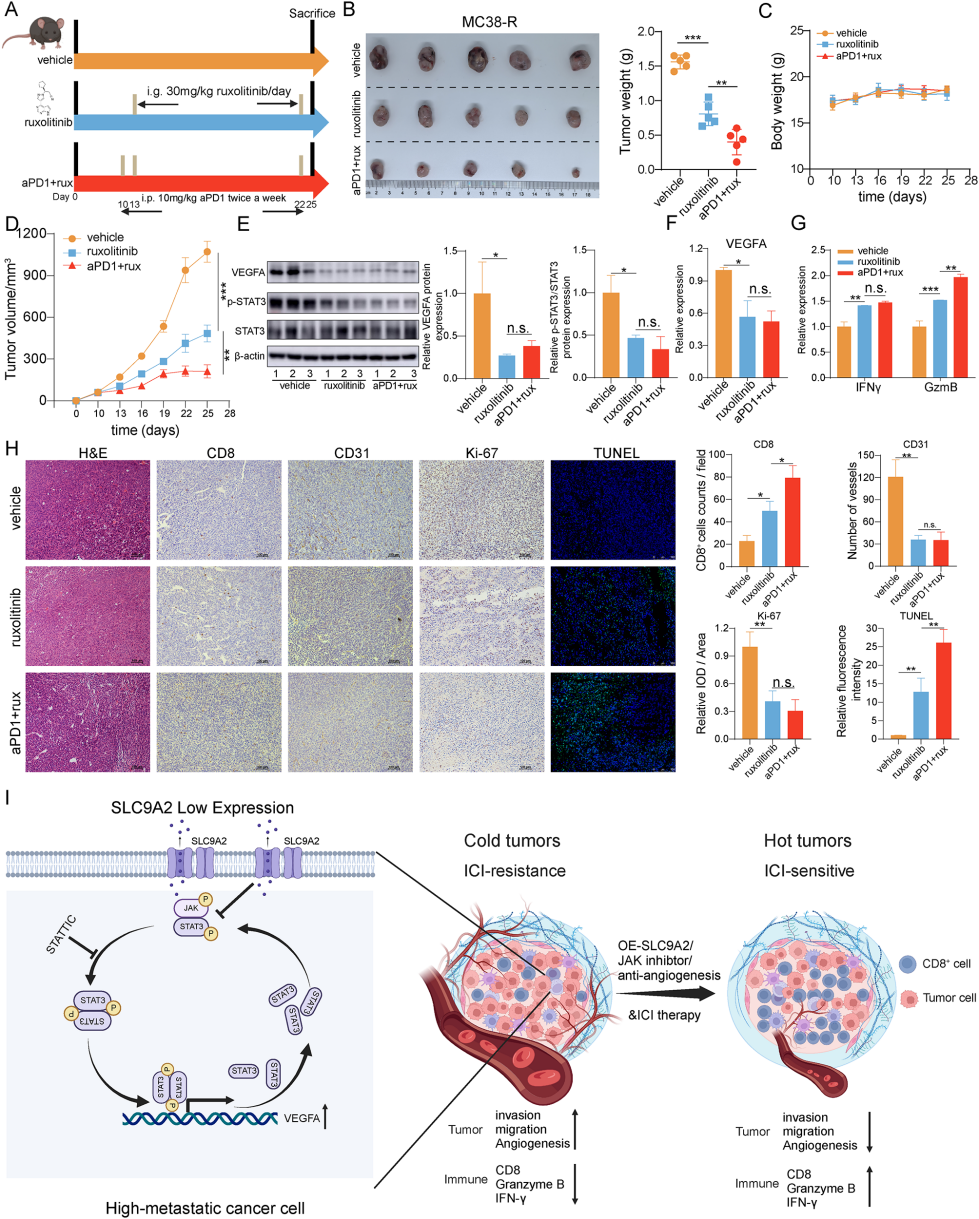
Ruxolitinib Augments the Effectiveness of Anti-PD-1 Immunotherapy against CRC. (**A**) MC38-R cells were used to establish subcutaneous tumor models in C57BL/6 mice. Starting on day 10, mice received intraperitoneal injections of anti-PD1, followed by oral administration of ruxolitinib starting on day 13. (**B-D**) Mice were sacrificed two weeks later, and tumors were harvested for photography and weight measurement (**B**). Tumor volume changes were analyzed for each group (**D**), along with assessments of body weight variations in the mice (**C**). (**E**) Western blot analysis was conducted to determine the levels of VEGFA and p-STAT3^Y705^ in the tumor tissues. (**F-G**) Levels of VEGFA (**F**), IFNγ, and Granzyme B (**G**) in tumor tissues from each group of mice were quantified using ELISA. The mean of the control group was normalized to 1 for statistical analysis and graphical representation. (**H**) Tumor tissues were embedded in paraffin and subjected to H&E staining, as well as immunohistochemical analysis for CD8, CD31, Ki-67 and TUNEL staining. (**I**) Diagram illustrating the proposed mechanism by which SLC9A2 inhibits immune evasion in CRC. Data in bar graphs indicate mean ± SEM, and data were analyzed using multi-group analysis of variance. *P < 0.05, **P < 0.01, ***P < 0.001, n.s., not significant

We found that a daily dose of 30 mg/kg Ruxolitinib was the most efficacious, significantly reducing tumor growth when combined with immune checkpoint inhibitors (ruxolitinib + ICI), while demonstrating a favorable safety profile with no hepatotoxicity, nephrotoxicity, or pulmonary toxicity. (Fig. 8A-D, Figure S3). Western blot analysis of intratumoral p-STAT3 and VEGFA levels revealed that Ruxolitinib effectively inhibited STAT3 phosphorylation and reduced VEGFA expression (Fig. 8E). Furthermore, ELISA assays confirmed a decrease in VEGFA secretion (Fig. 8F). ELISA results also validated that Ruxolitinib can stimulate granzyme B and IFN-γ production (Fig. 8G). Immunohistochemical analysis of CDX demonstrated that Ruxolitinib reduced the number of blood vessels and promoted CD8^+^ T cell infiltration (Fig. 8H). Following Ruxolitinib treatment, there was a reduction in Ki67-positive staining cells, accompanied by an increase in TUNEL^+^ apoptotic cells (Fig. 8H). These findings suggest that the combination of Ruxolitinib and anti-PD-1 therapy may have significant clinical implications for the treatment of CRC.

## Discussion

The management of metachronous liver metastasis in CRC poses a significant challenge within clinical practice. On one hand, this type of metastasis demonstrates limited responsiveness to systemic chemotherapy and current targeted therapies, such as 5-FU and anti-PD-1 [29]. On the other hand, effective molecular biomarkers to predict liver metastasis occurrence in postoperative CRC patients remain lacking. Thus, elucidating the molecular mechanisms underlying CRC liver metastasis is crucial for identifying novel therapeutic targets and screening predictive biomarkers.

In the current study, we established a highly metastatic CRC cell line and performed RNA-Seq. Through differential expression analysis of independent databases related to primary CRC and liver metastatic lesions, we identified SLC9A2 as the gene most strongly associated with CRC liver metastasis. Our findings indicate that SLC9A2 inhibits the migration and invasion capabilities of CRC cells, a result substantiated at the experimental level through splenic injection of CRC cells in a mouse model of liver metastasis. Furthermore, analyses of multiple independent databases confirmed a close association between downregulated SLC9A2 and both metachronous liver metastasis and poor prognosis in patients. Analysis of CRC immunotherapy cohorts revealed that patients with high SLC9A2 expression exhibited improved treatment responses. Establishing an MC38 anti-PD-1-resistant model demonstrated that intratumoral overexpression of Slc9a2 could reverse resistance to immunotherapy. Mechanistically, SLC9A2 suppresses the EMT pathway and subsequent metastasis by inhibiting STAT3^Y705^ while also reducing tumor cell secretion of VEGFA, thus limiting tumor angiogenesis. In early-stage CRC, SLC9A2 can inhibit tumor metastatic progression, while in advanced stages, it serves as an immunosensitizing molecule that reverses resistance to immunotherapy in liver metastasis. Collectively, our data underscore the critical regulatory role of SLC9A2 in CRC progression and its potential as a therapeutic target for CRC treatment.

Patients with high SLC9A2 expression may benefit from immunotherapy (e.g., anti-PD-1) alone, as their tumors are likely to have normalized vasculature and a “hot” immune microenvironment, which facilitates T-cell infiltration. In contrast, patients with low SLC9A2 expression—typically associated with advanced TNM stages, liver metastases, and resistance to immunotherapy—may require combinatorial treatment strategies. Our data suggest that combining anti-PD-1 with JAK/STAT3 inhibitors (e.g., Ruxolitinib) or anti-angiogenic agents (e.g., Bevacizumab) could help overcome resistance by inhibiting STAT3-driven VEGFA production and restoring immune cell function. Longitudinal monitoring of SLC9A2 expression during treatment could further refine therapeutic strategies. For example, the loss of SLC9A2 in recurrent or metastatic lesions may indicate the need for more aggressive anti-angiogenic or STAT3-targeted therapies. Additionally, emerging technologies such as liquid biopsies that detect SLC9A2 in circulating tumor cell could enable non-invasive tracking of biomarkers. Integrating SLC9A2 status with other molecular markers (e.g., MSI status, KRAS mutations) may further enhance precision oncology by identifying patients who are most likely to benefit from specific therapies.

However, targeting SLC9A2 as a therapeutic candidate presents considerable challenges. Firstly, as SLC9A2 is physiologically expressed in the gastrointestinal epithelia and kidneys, systemic therapies aimed at upregulating its expression could inadvertently disrupt ion transport or pH regulation in normal tissues. To address this, the development of CRC-specific delivery systems, such as nanoparticle-based platforms or tumor-targeted gene editing tools, may help minimize off-target effects. Moreover, since SLC9A2 functions as a tumor suppressor gene, it is critical to precisely control its expression levels within tumors to avoid potential toxicity associated with overexpression. Secondly, there are currently no approved drugs that directly modulate SLC9A2 activity. Future efforts should focus on developing small-molecule activators or CRISPR/dCas9-based strategies to restore its function. At the same time, indirect approaches, such as inhibiting the JAK/STAT3 pathway (e.g., using Ruxolitinib, as demonstrated in our study), could serve as potential interim solutions to mitigate oncogenic signaling related to SLC9A2 deficiency.

SLC9A2, also known as NHE2, is a protein-coding gene primarily expressed in the apical membrane of gastrointestinal epithelial cells. It encodes a member of NHE family, which is essential for ion transport and homeostasis [30]. Previous studies have established that SLC9A2 may serve as a promising biomarker for differentiating colon adenomatous polyps from colorectal carcinoma [31]. Furthermore, SLC9A2 has been shown to inhibit the growth and invasion of osteosarcoma through the suppression of aerobic glycolysis [32]. However, some studies have reported that SLC9A2 is involved in the development of drug resistance in ovarian cancer [33]. Consistent with our research, Liu et al. indicated that SLC9A2 inhibits the progression of CRC by downregulating the MAPK pathway [34]. Despite this, limited research exists on its impact on the tumor immune microenvironment and its effects on CRC immunotherapy.

Evidence increasingly suggests that CRC exhibits a distinct angiogenic phenotype, positioning angiogenesis as a viable therapeutic target [25]. Notably, our results demonstrated that SLC9A2 is highly expressed in primary CRC tumors but shows reduced expression in liver metastatic lesions. In contrast, microvessel density is inversely correlated, with low expression in primary CRC and high expression in liver metastases, indicating a negative correlation between SLC9A2 expression and microvessel density in clinical CRC samples. Further in vivo and in vitro experiments confirmed the inhibition of angiogenesis by SLC9A2. Currently, the mechanisms by which SLC9A2 promotes tumor angiogenesis have not been extensively studied. VEGFA is a key angiogenic factor in tumors, playing a significant role in the initial stages of tumor development, progression, and metastasis [35]. Correlation analyses revealed a negative relationship between SLC9A2 and VEGFA, with our findings indicating that SLC9A2 downregulates VEGFA. VEGFA is crucial for enhancing tumor angiogenesis, promoting migration and invasion, and contributing to the formation of an immunosuppressive tumor microenvironment. However, the relationship between SLC9A2 and VEGFA remains largely unclear. Our study demonstrated that VEGFA expression decreased with SLC9A2 overexpression and increased with SLC9A2 silencing.

Transcription factors play a critical role in regulating VEGFA expression, with STAT3 recognized for its ability to activate VEGFA in cancer cells [36, 37]. STAT3 is a member of the STAT family of transcription factors and becomes phosphorylated by JAK, forming its active form, pSTAT3 (Tyr705) [38]. This phosphorylated form dimerizes, translocates to the nucleus, and binds to the promoter of target genes, promoting their expression [39]. Our findings indicate that downregulation of SLC9A2 significantly enhances STAT3 phosphorylation, whereas overexpression of SLC9A2 markedly reduces both STAT3 phosphorylation and nuclear localization in CRC cells. Moreover, the inhibition of STAT3 phosphorylation completely reverses the effects of SLC9A2 knockdown on VEGFA expression and angiogenesis, both in vivo and in vitro. These results align with previous studies demonstrating VEGF-independent angiogenesis through STAT3 signaling. Therefore, angiogenesis induced by SLC9A2 downregulation in CRC appears to depend on the STAT3/VEGFA pathway. Additionally, these data suggest that SLC9A2 has the potential to serve as a biomarker for anti-VEGF-targeted angiogenesis therapy in CRC.

Unfortunately, CRC patients receiving anti-angiogenic therapy as a sole treatment do not exhibit significant improvements in clinical outcomes [40, 41]. Aberrant vasculature is a hallmark of most solid tumors and facilitates immune evasion [42]. Increasing evidence suggests that modulating angiogenesis within the tumor microenvironment can enhance resistance to immunotherapy. Clinical trials have shown that anti-VEGF treatment creates a window for tumor vascular normalization, thereby increasing the recruitment of immune cells into the tumor immune microenvironment and enhancing the tumor-killing capabilities of these cells [43]. Consequently, patients with advanced CRC should consider combination therapies involving immunotherapy and anti-angiogenic treatment [42]. However, prolonged VEGF blockade can lead to increased tumor hypoxia, resulting in resistance to hypoxia-induced apoptosis and elevated VEGF expression, thus promoting tumor aggressiveness [44, 45]. This may explain the failures observed in many clinical trials, including those involving anti-angiogenic therapies combined with chemotherapy or immunotherapy [46]. Unlike Bevacizumab (a humanized IgG1 monoclonal antibody targeting VEGFA), our study indicates that SLC9A2 can inhibit VEGFA synthesis, effectively blocking VEGFA production at its source and serving as a superior target for anti-tumor angiogenesis strategies.

In addition to its role in angiogenesis, SLC9A2 also suppresses tumor cell metastasis and invasion by downregulating the STAT3 signaling pathway, thereby promoting the EMT process in CRC. Transcription factors STAT1, STAT3, and STAT6 are involved in signaling downstream of JAK1 and JAK2. JAK inhibition has been shown to enhance checkpoint blockade immunotherapy in patients with non-small cell lung cancer and Hodgkin lymphoma [26, 27]. Mathew et al. reported that JAK inhibition may particularly benefit patients with elevated inflammation and poor CD8^+^ T cell responses to anti–PD-1 therapy alone [26]. Furthermore, Zak et al. demonstrated that small-molecule JAK inhibition synergizes with checkpoint blockade immunotherapy to reshape the myeloid cell compartment, enhancing natural killer (NK) and T cell responses [27]. Thus, the JAK inhibitor Ruxolitinib not only effectively suppresses the JAK-STAT3 signaling pathway within tumors, inhibiting both tumor metastasis and angiogenesis, but also significantly activates the tumor immune microenvironment, facilitating a transition from a “cold” to a “hot” immune landscape. This multifaceted mechanism of action allows Ruxolitinib to achieve robust anti-tumor effects.

In conclusion, our study reveals the critical involvement of SLC9A2 in tumor metastasis and angiogenesis, uncovering a previously unrecognized mechanism by which SLC9A2 facilitates the dephosphorylation of STAT3, thereby suppressing the oncogenic effects of STAT3 signaling in CRC (Fig. 8I). Additionally, we found that SLC9A2 enhances the efficacy of immunotherapy by targeting tumor angiogenesis. Furthermore, our research identifies the JAK inhibitor Ruxolitinib as a promising agent that can inhibit tumor metastasis at early stages and synergize with immunotherapy in advanced stages, particularly benefiting patients with low SLC9A2 expression.

## Electronic Supplementary Material

Below is the link to the electronic supplementary material.

Supplementary Material 1

## Data Availability

No datasets were generated or analysed during the current study.

## Abbreviations

CRC: Colorectal cancer
NHE: Na^+^/H^+^ exchanger
VEGFA: Vascular Endothelial Growth Factor A
HUVECS: Human Umbilical Vein Endothelial Cells
FBS: Fetal bovine serum
SPF: Specific pathogen-free
PVDF: Polyvinylidene difluoride
EdU: 5-Ethynyl-2′-Deoxyuridine
RT: Room temperature
CM: Conditional medium
PDOs: Patient derived organoids
RT-qPCR: Reverse transcription quantitative real-time PCR
NOD-SCID: NOD.CB17-Prkdc scid /NcrCrl
H&E: Hematoxylin and eosin
IHC: Immunohistochemistry
FFPE: Formalin-fixedparaffin-embedded
IF: Immunofluorescence
TCGA: The Cancer Genome Atlas
COAD: Colon adenocarcinoma
READ: Rectum adenocarcinoma
GEO: Gene Expression Omnibus
TISCH: Tumor Immune Single-cell Hub
CancerSEA: Cancer Single-cell State Atlas
EMT: Epithelial-mesenchymal transition
OS: Overall survival
RFS: Recurrence-free survival
DSS: Disease-specific survival
PFS: Progression-free survival
MSI-H: Microsatellite instability-high
MSS: Microsatellite stable
GSEA: Gene Set Enrichment Analysis
KEGG: Kyoto Encyclopedia of Genes and Genomes
GO: Gene Ontology
CDX: Cell line-derived xenografts
TUNEL: Terminal deoxynucleotidyl transferase dUTP nick end labeling
NK: Natural killer
