# Supplementary material for: Tumor suppressor SLC9A2 inhibits colorectal cancer metastasis and reverses immunotherapy resistance by suppressing angiogenesis
Source: J Exp Clin Cancer Res. 2025 Jun 5;44:172. doi: 10.1186/s13046-025-03422-7 (PMC12139321; doi:10.1186/s13046-025-03422-7)
Supplement: Supplementary file 1 — Supplementary Material 1 [file 13046_2025_3422_MOESM1_ESM.docx]

**Supplementary material for “Tumor suppressor SLC9A2 inhibits colorectal cancer metastasis and reverses immunotherapy resistance by suppressing angiogenesis”**

**Figure. S1**


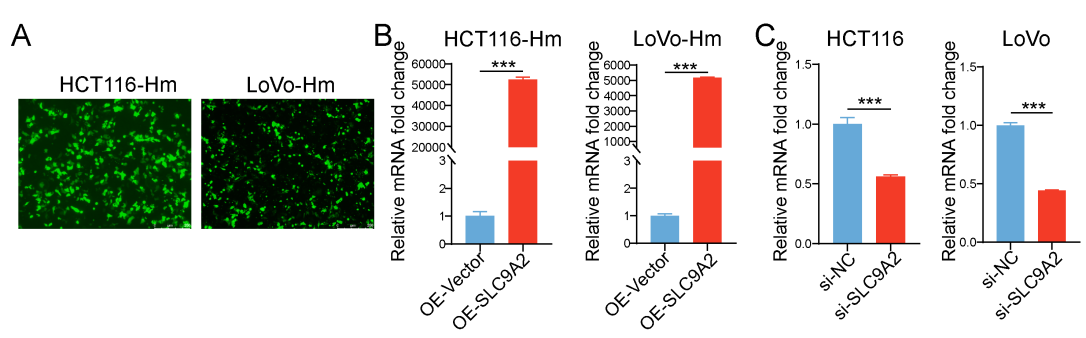


**Figure S1 Assessment of SLC9A2 expression efficiency in CRC cells.** (A-B) Fluorescent images of HCT116-Hm and LoVo-Hm cells stably expressing SLC9A2 were obtained (A), and RT-qPCR was conducted to assess transcriptional levels (B). (C) RT-qPCR analysis of SLC9A2 knockdown efficiency in HCT116 and LoVo cells. Data in bar graphs indicate mean ± SEM, and data were analyzed using Student’s t test. ****P* < 0.001.

**Figure. S2**


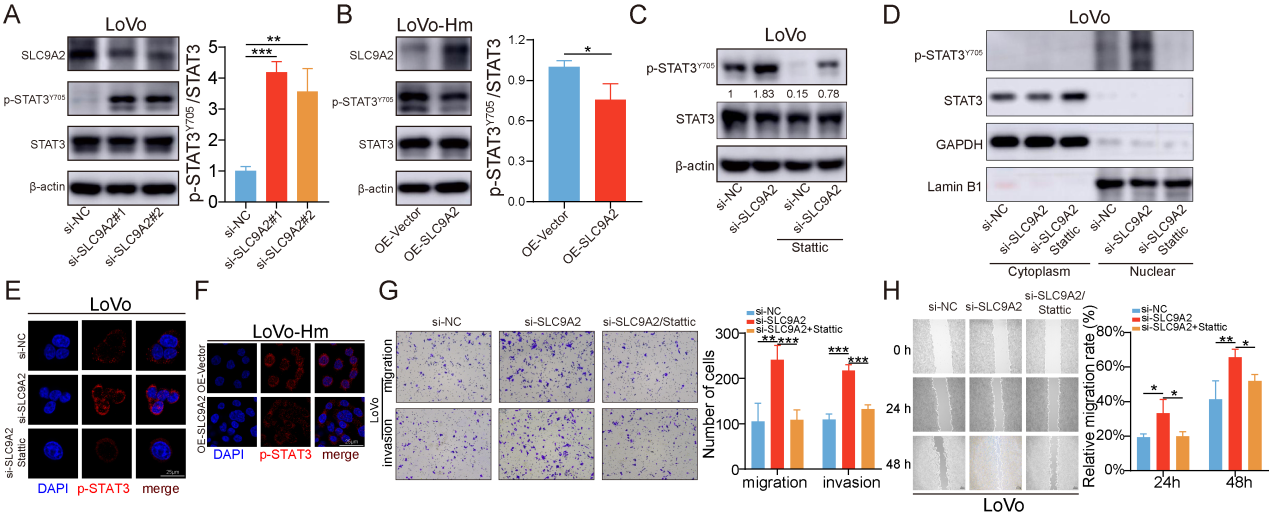


**Figure S2 Impact of SLC9A2 on CRC cell metastasis.** (A) Western blot analysis was performed to measure the levels of SLC9A2, STAT3 and p-STAT3^Y705^ in LoVo cells transfected with either siRNA-NC or siRNA-SLC9A2. (B) Western blot analysis was performed to measure the levels of SLC9A2, STAT3 and p-STAT3^Y705^ in LoVo-Hm cells overexpressing either Vector or SLC9A2. (C) LoVo cells were transfected with either siRNA-NC or siRNA-SLC9A2, followed by treatment with or without 5 µM Stattic for 24 hours. After 48 hours, Western blot analysis was performed to evaluate the levels of STAT3 and p-STAT3^Y705^. (D) LoVo cells were transfected with either siRNA-NC or siRNA-SLC9A2, and the siRNA-SLC9A2 group was subsequently treated with 5 µM Stattic for 24 hours. Following a 48-hour incubation, nuclear-cytoplasmic separation was performed, and Western blot analysis was conducted to evaluate the levels of STAT3 and p-STAT3^Y705^. (E) LoVo cells were transfected with either siRNA-NC or siRNA-SLC9A2, and the siRNA-SLC9A2 group was subsequently treated with 5 µM Stattic for 24 hours. Following a 48-hour incubation, immunofluorescence was used to assess the nuclear localization of p- STAT3^Y705^. (F) LoVo-Hm cells were transfected with either vector or SLC9A2. After 24 hours, immunofluorescence was used to examine the nuclear localization of p-STAT3^Y705^. (G) Migration and invasion assays were conducted on LoVo cells transfected with either siRNA-NC or siRNA-SLC9A2, and the siRNA-SLC9A2 group was subsequently treated with 5 µM Stattic for 24 hours. (H) Wound healing assay was conducted on LoVo cells transfected with either siRNA-NC or siRNA-SLC9A2, and the siRNA-SLC9A2 group was subsequently treated with 5 µM Stattic for 24 hours. Data in bar graphs indicate mean ± SEM. **P* < 0.05, ***P* < 0.01, ****P* < 0.001. Multi-group analysis of variance (A, G, H), Student’s t test (B).

**Figure. S3**


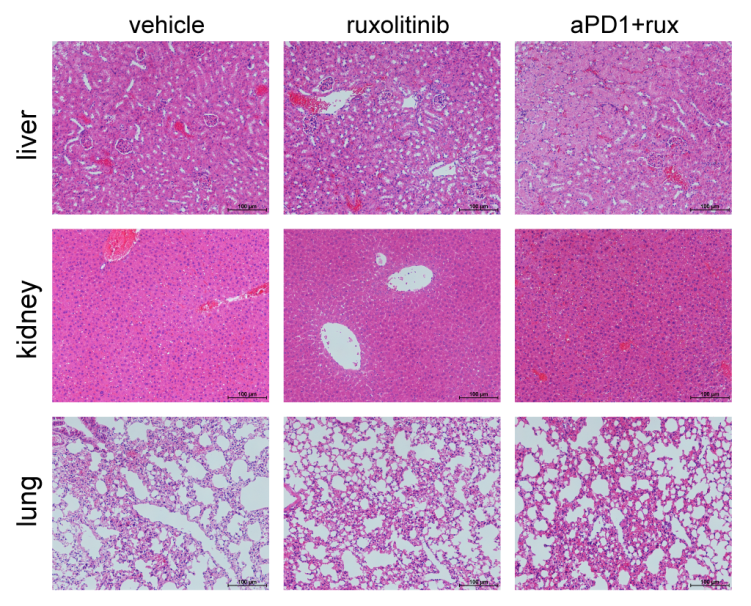


**Figure S3** H&E staining showed that ruxolitinib had no obvious toxic effects on the liver, kidney, lung *in vivo*.

**Table. S1：Human primers for RT-qPCR**

| Primers name | Sequence (5’ - 3’) |
| --- | --- |
| ACTB-F | GAACGGTGAAGGTGACAGCA |
| ACTB-R | GTAACAACGCATCTCATATTTGGAA |
| SLC9A2-F | CAGATCCCCTTCGAGATCACC |
| SLC9A2-R | GGGAGACTTCTCATCAACACCAA |
| CDH1-F | CGAGAGCTACACGTTCACGG |
| CDH1-R | GGGTGTCGAGGGAAAAATAGG |
| CDH2-F | TGCGGTACAGTGTAACTGGG |
| CDH2-R | GAAACCGGGCTATCTGCTCG |
| VIM-F | GACGCCATCAACACCGAGTT |
| VIM-R | CTTTGTCGTTGGTTAGCTGGT |
